# Opposing transcriptional programs of KLF5 and AR emerge during therapy for advanced prostate cancer

**DOI:** 10.1038/s41467-021-26612-1

**Published:** 2021-11-04

**Authors:** Meixia Che, Aashi Chaturvedi, Sarah A. Munro, Samuel P. Pitzen, Alex Ling, Weijie Zhang, Josh Mentzer, Sheng-Yu Ku, Loredana Puca, Yanyun Zhu, Andries M. Bergman, Tesa M. Severson, Colleen Forster, Yuzhen Liu, Jacob Hildebrand, Mark Daniel, Ting-You Wang, Luke A. Selth, Theresa Hickey, Amina Zoubeidi, Martin Gleave, Rohan Bareja, Andrea Sboner, Wayne Tilley, Jason S. Carroll, Winston Tan, Manish Kohli, Rendong Yang, Andrew C. Hsieh, Paari Murugan, Wilbert Zwart, Himisha Beltran, R. Stephanie Huang, Scott M. Dehm

**Affiliations:** 1 Masonic Cancer Center, University of Minnesota, Minneapolis, MN 55455 USA; 2 University of Minnesota Supercomputing Institute, University of Minnesota, Minneapolis, MN 55455 USA; 3 Graduate Program in Molecular, Cellular, and Developmental Biology and Genetics, University of Minnesota, Minneapolis, MN 55455 USA; 4 Department of Experimental and Clinical Pharmacology, University of Minnesota, Minneapolis, MN 55455 USA; 5 Department of Medical Oncology, Dana Farber Cancer Institute and Harvard Medical School, Boston, MA 02215 USA; 6 Division of Medical Oncology, Weill Cornell Medicine, New York, NY 10065 USA; 7 Division on Oncogenomics, Oncode Institute, The Netherlands Cancer Institute, Amsterdam, The Netherlands; 8 Department of Laboratory Medicine and Pathology, University of Minnesota, Minneapolis, MN 55455 USA; 9 Divisions of Human Biology and Clinical Research, Fred Hutchinson Cancer Research Center, Seattle, WA 98109 USA; 10 Graduate Program in Microbiology, Immunology, and Cancer Biology, University of Minnesota, Minneapolis, MN 55455 USA; 11 The Hormel Institute, University of Minnesota, Austin, MN 55912 USA; 12 Flinders Health and Medical Research Institute and Flinders Centre for Innovation in Cancer, Flinders University, Bedford Park, SA Australia; 13 Dame Roma Mitchell Cancer Research Laboratories and Freemasons Foundation Centre for Men’s Health, Adelaide Medical School, The University of Adelaide, Adelaide, SA Australia; 14 Department of Urologic Sciences, University of British Columbia, Vancouver, BC V5Z 1M9 Canada; 15 Vancouver Prostate Centre, Vancouver, BC V6H 3Z6 Canada; 16 Englander Institute for Precision Medicine, Weill Cornell Medicine, New York, NY 10065 USA; 17 Cancer Research UK, University of Cambridge, CB2 0RE Cambridge, UK; 18 Department of Medicine, Mayo Clinic, Jacksonville, FL 32224 USA; 19 Huntsman Cancer Institute, University of Utah, Salt Lake City, UT 84112 USA; 20 Department of Urology, University of Minnesota, Minneapolis, MN 55455 USA

**Keywords:** Transcription factors, Prostate cancer

## Abstract

Endocrine therapies for prostate cancer inhibit the androgen receptor (AR) transcription factor. In most cases, AR activity resumes during therapy and drives progression to castration-resistant prostate cancer (CRPC). However, therapy can also promote lineage plasticity and select for AR-independent phenotypes that are uniformly lethal. Here, we demonstrate the stem cell transcription factor Krüppel-like factor 5 (KLF5) is low or absent in prostate cancers prior to endocrine therapy, but induced in a subset of CRPC, including CRPC displaying lineage plasticity. KLF5 and AR physically interact on chromatin and drive opposing transcriptional programs, with KLF5 promoting cellular migration, anchorage-independent growth, and basal epithelial cell phenotypes. We identify *ERBB2* as a point of transcriptional convergence displaying activation by KLF5 and repression by AR. ERBB2 inhibitors preferentially block KLF5-driven oncogenic phenotypes. These findings implicate KLF5 as an oncogene that can be upregulated in CRPC to oppose AR activities and promote lineage plasticity.

## Abstract (web summary)

While many treatments for prostate cancer suppress the androgen receptor it becomes reactivated during disease progression. Here, the authors show that a KLF5 transcriptional programme is also activated during treatment and promotes migration and the appearance of a basal cell phenotype.

## Introduction

The human prostate contains glandular acini composed of luminal epithelial cells, basal epithelial cells, and rare interspersed neuroendocrine epithelial cells, all supported by a surrounding stroma^1^. Testes-derived androgens testosterone or dihydrotestosterone (DHT) bind and activate the AR, a transcription factor that regulates homeostasis of prostate luminal cells^2^. Castration-induced androgen depletion inhibits AR activity, causing apoptosis of luminal cells and prostate regression^2^. Most prostate cancer cells have a luminal identity and remain dependent on AR activity^3^. Therefore, castration-based therapies are effective treatments for advanced prostate cancer^4^.

Prostate basal cells are AR-negative and insensitive to androgen depletion^3^. The Krüppel-like factor 5 (KLF5) transcription factor is functionally redundant with the Yamanaka factor KLF4 for maintaining embryonic stem cell self-renewal^5^. *Klf5* deletion in the mouse prostate reduces basal cell populations, including rare basal cells with stem/progenitor properties^6^. Co-deletion of *Klf5* and *Pten* in the mouse prostate accelerates tumorigenesis compared with *Pten* deletion alone, indicating that KLF5 is a prostate tumor suppressor^7^. Consistent with this notion, KLF5 protein expression is low or absent in prostate cancer relative to normal prostate, which is due to *KLF5* gene deletion in a subset of cases^8,9^. Intriguingly, in bladder, intestinal, breast, and gastric cancers, KLF5 is oncogenic and linked to poor prognosis^10–13^, often due to activating *KLF5* gene alterations^14^. The molecular mechanisms that govern KLF5 functioning as a tumor suppressor in prostate cancer, and an oncogenic factor in other cancers, are currently unknown.

A challenge in prostate cancer management is the inevitable emergence of castration-resistant prostate cancer (CRPC) during androgen deprivation therapy. In most cases, CRPC is driven by AR re-activation^15^, and more potent AR-targeted therapies, including abiraterone acetate and enzalutamide, are effective. However, resistance ultimately limits the therapeutic durability of abiraterone and enzalutamide. Additionally, the selective pressures exerted by these potent therapies can promote lineage plasticity where transcriptional and epigenetic regulators are upregulated to support non-luminal, AR-independent cell phenotypes such as neuroendocrine CRPC (NEPC)^16–20^. However, mechanisms in the lineage plasticity cascades that initiate early steps in luminal de-differentiation are poorly defined. Here, we show that KLF5 is expressed at high levels in a subset of CRPC tissues and models, including NEPC. In this context, KLF5 supports oncogenic phenotypes and transcriptionally opposes AR to induce ERBB2 and basal cell identity. These findings indicate that castration-based therapies switch KLF5 from a tumor suppressor to an oncogene, thereby promoting luminal cell de-differentiation during CRPC progression.

## Results

### Transcriptional up-regulation of KLF5 in a subset of CRPC

**Fig. 1:**
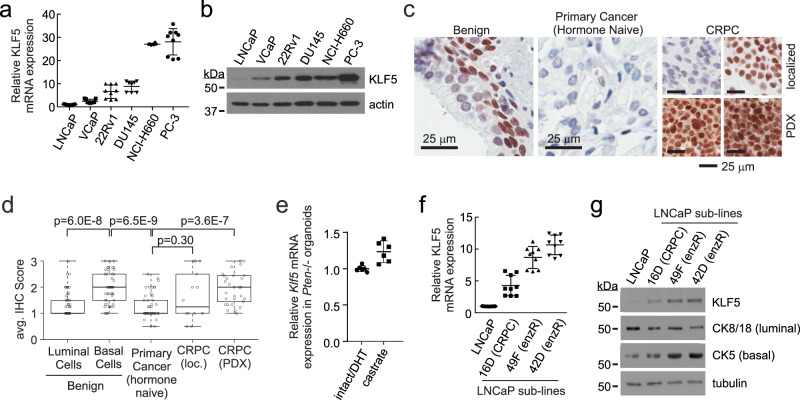
KLF5 levels are upregulated in CRPC. **a** *KLF5* mRNA measured using quantitative RT-PCR in AR-positive LNCaP, VCaP, 22Rv1 and AR-negative DU145, NCI-H660 and PC-3 cell lines. *n* = 9, mean ± 95% CI from three biological replicates in technical triplicate. **b** Protein measured by western blot using the same cell lines as **a**. Actin is a loading control. **c** Representative immunohistochemistry (IHC) staining of benign, localized prostate cancer (hormone naïve) and CRPC tissues from patients or patient-derived xenografts (PDX) using an antibody specific for KLF5. **d** Quantification of KLF5 IHC staining in tissue microarrays by a genitourinary pathologist (PM). Bounds of boxes are lower/upper quartiles with median; whiskers show range from minima to maxima; dots are the average staining intensity of an individual case or PDX. *n* = 50 localized prostate cancer cases (*n* = 50/50 cases contained benign luminal cells, *n* = 47/50 cases contained benign basal cells, n = 47/50 slides contained cancer cells), *n* = 14 localized CRPC cases, and *n* = 29 CRPC PDXs. *P*-values are from two-sided Mann–Whitney *U*-tests. n.s. = not significant. **e** *Klf5* mRNA measured using quantitative RT-PCR in organoids derived from intact *Pten*−/− mouse prostates maintained in androgen-replete (DHT, dihydrotestosterone) conditions or castrate *Pten*−/− mouse prostates maintained in androgen-depleted conditions. *n* = 6, mean ±95% CI from two biological replicates in technical triplicate. **f** *KLF5* mRNA measured by RT-PCR in LNCaP cells and sub-lines derived from castration-resistant (16D) or castration/enzalutamide-resistant (49F and 42D) LNCaP xenograft tumors. *n* = 9, mean ±195% CI from three biological replicates in technical triplicate. **g** Protein measured by western blot using the same cell lines as in **f**. Tubulin is a loading control.

We evaluated KLF5 expression at various stages of prostate cancer progression. KLF5 mRNA and protein levels were low in AR-positive, androgen-dependent cell lines LNCaP and VCaP, intermediate in AR-positive CRPC 22Rv1 cells, and high in AR-negative CRPC cell lines DU145, NCI-H660, and PC-3 (Fig. 1a, b). KLF5 staining was high in basal epithelial cells from benign prostate tissue and low in luminal epithelial cells (Fig. 1c, d). Lower KLF5 staining occurred in localized prostate cancer relative to benign prostate, which is consistent with loss of basal cells and expansion of luminal cells during tumorigenesis^3^. In CRPC tissues obtained from clinical procedures or CRPC tissues propagated as patient-derived xenografts (PDXs), KLF5 staining ranged from very low to intense, with average KLF5 levels in CRPC PDXs being higher than observed in localized prostate cancer.

We next evaluated whether AR-targeted therapies affected KLF5 expression. *Klf5* mRNA levels in organoids from prostates of castrated *Pten* −/− mice were 25% higher than in organoids from prostates of intact *Pten* −/− mice (Fig. 1e)^21^. Further, KLF5 mRNA and protein levels were higher in LNCaP sub-lines derived from xenografts that had progressed in castrated mice to a CRPC phenotype (16D) or an enzalutamide-resistant CRPC phenotype (49F and 42D)^16^ (Fig. 1f, g). In LNCaP 16D, 49F, and 42D cells, levels of the basal cell marker CK5 were also higher and levels of the luminal cell markers CK8/18 were lower. KLF5 mRNA and protein levels were also elevated in the LNCaP95 cell line derived from the long-term passage of LNCaP cells in an androgen-depleted medium^22^ (Supplementary Fig. 1). These data demonstrate that upregulation of KLF5 occurs in prostate cancer subjected to AR inhibition in vitro and in vivo.

### AR inhibition promotes durable KLF5 transcriptional activation by androgens

**Fig. 2:**
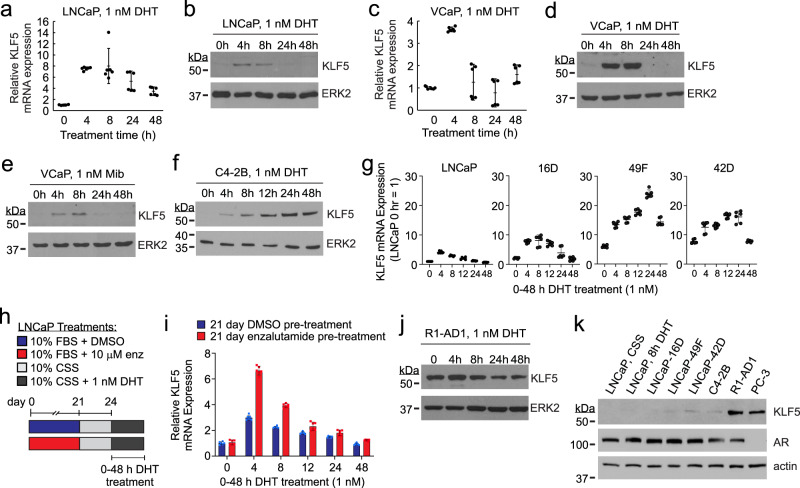
CRPC models display increased durability of KLF5 induction by androgens. **a** *KLF5* mRNA measured by RT-PCR in LNCaP cells cultured in androgen-deplete medium supplemented with 1 nM DHT for indicated time-points. *n* = 6, mean ± 95% CI from 2 biological replicates in technical triplicate. **b** KLF5 protein measured by western blot using the same conditions as **a**. ERK2 is a loading control. **c** *KLF5* mRNA measured by RT-PCR in VCaP cells as in **a**. *n* = 6, mean ± 95% CI from 2 biological replicates in technical triplicate. **d** KLF5 protein measured by western blot using the same conditions as in **c**. ERK2 is a loading control. **e** KLF5 protein measured by western blot in VCaP cells treated with 1 nM mibolerone (Mib) as in **d**. One additional replicate experiment was performed that yielded a comparable result. **f** KLF5 protein measured by western blot in C4-2B cells treated as in **b**. One additional replicate experiment was performed that yielded a comparable result. **g** *KLF5* mRNA measured by RT-PCR in LNCaP and LNCaP-derived CRPC sub-lines 16D, 49F, and 42D cells treated as in **a**. *n* = 6, mean ± 95% CI from three biological replicates in technical duplicate. **h** Schematic of LNCaP cell culture conditions for **i**. enz = enzalutamide, DMSO = vehicle control. **i** *KLF5* mRNA measured by RT-PCR in cells cultured as in **i**. *n* = 6, mean ± 95% CI from 2 biological replicates in technical triplicate. **j** KLF5 protein measured in R1-AD1 cells by western blot using the same conditions as **a**. One additional replicate experiment was performed that yielded a comparable result. **k** KLF5 and AR proteins measured by western blot using LNCaP cells cultured in androgen-deplete medium (CSS), LNCaP cells stimulated 8 h with 1 nM DHT as in **a**, or indicated cell lines grown in their respective standard medium conditions. Actin is loading control. One additional replicate experiment was performed that yielded a comparable result.

Because KLF5 levels were highest in AR-null CRPC cells, we tested whether AR represses KLF5. However, the expression of AR in PC-3 and DU145 cells did not affect KLF5 levels (Supplementary Fig. 2a, b). Next, we considered prior reports of KLF5 induction by androgens^23–25^ and evaluated KLF5 regulation by AR in LNCaP and VCaP cells. KLF5 mRNA and protein levels were induced after 4–8 h exposure to 1 nM DHT but returned to baseline within 24 h (Fig. 2a–d, Supplementary Fig. 2c). This transient induction by androgens required AR (Supplementary Fig. 2d) and was not due to DHT metabolism because 1 nM doses of synthetic androgens produced similar responses (Fig. 2e and Supplementary Fig. 2e).

Transient induction of KLF5 by androgens was inconsistent with a recent report showing sustained induction of KLF5 in LNCaP and C4-2B cells treated with supraphysiological concentrations of androgens^25^. We found that a physiological 1 nM dose of DHT sustained KLF5 induction over 24–48 h in C4-2B cells (Fig. 2f). However, in LNCaP cells, sustained induction of KLF5 over 24 h was only observed when cells were treated with 10 nM of the synthetic androgen R1881 (Supplementary Fig. 2e). To explain the lowered threshold for sustained induction of KLF5 by androgens in C4-2B cells, we considered the fact that C4-2B represented a CRPC sub-line derived from LNCaP^26^ and hypothesized that prior exposure to castration could influence the transcriptional responsiveness of KLF5. To test this, we treated LNCaP, 16D, 49F, and 42D cells with 1 nM DHT. In addition to having higher baseline levels of KLF5, the CRPC 16D and enzalutamide-resistant 49F and 42D cell lines displayed a longer duration of KLF5 induction by androgens compared with parental LNCaP cells (Fig. 2g).

To directly test whether AR-targeted therapy enhances the transcriptional response of KLF5 to androgens, we cultured LNCaP cells in the enzalutamide-containing medium for 21 days, followed by a 3 day washout and subsequent treatment with 1 nM DHT (Fig. 2h). Enzalutamide pre-treatment did not affect the baseline levels of KLF5 but did cause a higher and more sustained induction of KLF5 by androgens than pre-treatment with vehicle (Fig. 2i).

We also tested baseline and androgen-induced levels of KLF5 in AR-positive R1-AD1 cells, which are androgen- and antiandrogen-sensitive cells derived from a CRPC CWR22 xenograft^27^. KLF5 displayed a modest induction by 1 nM DHT at 4 h, and returned to baseline levels by 8–24 h of treatment (Fig. 2j). Notably, the baseline level of KLF5 in R1-AD1 cells was comparable to PC-3 cells and much higher than LNCaP cells and LNCaP-derived models (Fig. 2k). Collectively, these findings confirm and extend published literature showing positive regulation of KLF5 by AR^23–25^. However, these findings also show that additional mechanisms contribute to KLF5 overexpression in CRPC.

### The *KLF5* super-enhancer displays different epigenetic states in prostate cancer cell lines

**Fig. 3:**
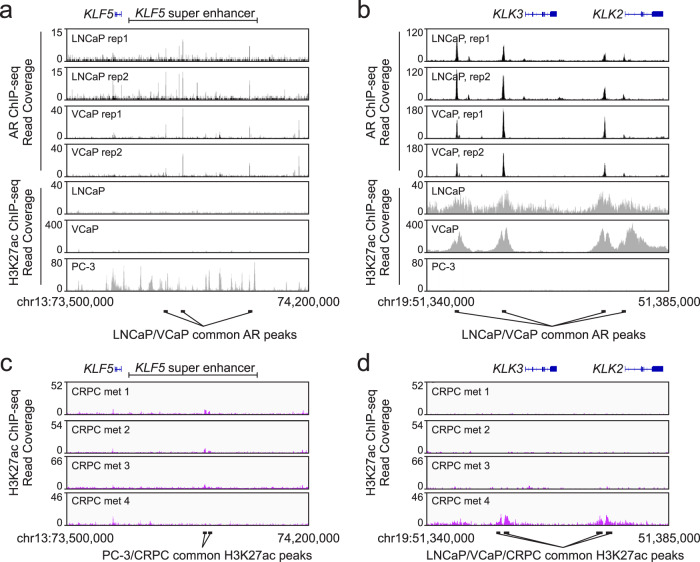
The *KLF5* and *KLK2/3* super-enhancers display an inverse pattern of H3K27ac activation marks. **a**, **b** Gene track views of H3K27ac and AR ChIP-seq data from AR-positive LNCaP and VCaP cell lines and the AR-negative PC-3 cell line at genomic loci for *KLF5* and *KLK2*/*KLK3*. **c**, **d** Gene track views of H3K27ac from four clinical CRPC specimens at genomic loci for *KLF5* and *KLK2*/*KLK3*. H3K27ac track heights were set using H3K27ac density at housekeeping gene enhancers shown in Supplementary Fig. 3.

To investigate androgen-independent mechanisms regulating KLF5 upregulation in CRPC, we evaluated a super-enhancer of the *KLF5* gene that displays genomic duplication and active chromatin marks in cancers where KLF5 is oncogenic^14,28^. AR binding within the *KLF5* super-enhancer was evident in chromatin immunoprecipitation DNA-sequencing (ChIP-seq) data from LNCaP and VCaP cells under androgen-replete conditions (Fig. 3a). However, the *KLF5* super-enhancer was devoid of ChIP-seq peaks for active enhancer marks histone H3 acetylated on lysine 27 (H3K27ac) or histone H3 methylated on lysine 4 (H3K4me1) relative to control enhancers near the *GAPDH*, *ACTB*, and *TUBA1A-C* genes (Fig. 3a, Supplementary Fig. 3a–h). Conversely, KLF5-high PC-3 cells displayed a high density of peaks for H3K27ac and H3K4me1 across the *KLF5* super-enhancer. This inverse relationship between H3K27ac/H3K4me1 density and AR binding in the *KLF5* super-enhancer contrasted with the *KLK2*/*KLK3* super-enhancer, where AR binding and H3K27ac/H3K4me1 density were strongly correlated (Fig. 3b, Supplementary Fig. 3e).

In CRPC patient metastases with low H3K27ac density in the *KLK2*/*KLK3* super-enhancer, the *KLF5* super-enhancer displayed H3K27ac peaks that overlapped those in PC-3 cells (Fig. 3c, d, Supplementary Fig. 3i–k). These *KLF5* super-enhancer H3K27ac peaks were absent in a CRPC metastasis that displayed high H3K27ac density in the *KLK2*/*KLK3* super-enhancer (compare CRPC met 4 to CRPC mets 1–3 in Fig. 3c, d and control enhancers in Supplementary Fig. 3i–k). These data demonstrate that PC-3 cells and CRPC tumors with reduced/lost H3K27ac and/or AR at the *KLK2*/*KLK3* super-enhancer display overlapping H3K27ac peaks within the *KLF5* super-enhancer.

### KLF5 overexpression is oncogenic in AR-positive prostate cancer cells

**Fig. 4:**
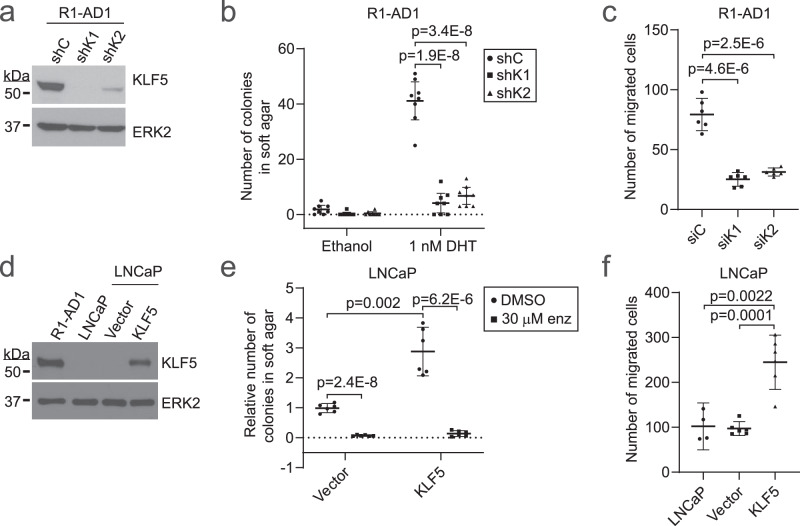
KLF5 promotes oncogenic phenotypes in prostate cancer cells. **a** KLF5 protein measured by western blot using R1-AD1 cells infected with control shRNA lentivirus (shC) or two independent KLF5-targeted shRNA lentiviruses (shK1 and shK2). ERK2 is a loading control. **b** R1-AD1 cells as in **a** were analyzed by 3D soft agar colony formation assays in androgen-deplete medium supplemented with 1 nM DHT or vehicle control (0.1% ethanol). *n* = 8, mean ±95% CI from 2 independent experiments in biological quadruplicate (*n* = 8). **c** R1-AD1 cells transfected with KLF5-targeted siRNAs (siK1 and siK2) or control siRNA (siC) were analyzed by chemotactic migration assays. *n* = 6, mean ±95% CI, 2 independent experiments in biological triplicate. **d** KLF5 protein measured by western blot using R1-AD1 cells, LNCaP cells, and LNCaP cells infected with empty lentivirus or lentivirus encoding KLF5. ERK2 is a loading control. **e** LNCaP cells as in **d** were analyzed by 3D soft agar colony formation assays in an androgen-replete medium supplemented with enzalutamide or vehicle control (DMSO). *n* = 6, mean ±95% CI, 2 independent experiments in biological triplicate. **f** LNCaP cells as in **d** were analyzed by chemotactic migration assays. *n* = 4 or 6, mean ±95% CI, 2 independent experiments in biological duplicate or triplicate. *P*-values are from 2-sided, 2-tailed *t*-tests.

A previous study demonstrated oncogenic effects of high KLF5 levels in AR-negative PC-3 and DU145 cells^29^. To investigate the effects of high KLF5 levels in an AR-positive context, we used the AR/KLF5-high R1-AD1 cell line. KLF5 knockdown reduced colony formation of R1-AD1 cells in soft agar under androgen-replete conditions (Fig. 4a, b) and reduced R1-AD1 cell migration towards a serum gradient in trans-well migration assays (Fig. 4c). However, KLF5 knockdown only modestly reduced 2-dimensional (2D) growth under androgen-depleted conditions but not androgen-replete conditions (Supplementary Fig. 4a, b). Overexpression of KLF5 in LNCaP cells increased 2D growth under androgen-depleted and androgen-replete conditions (Fig. 4d, Supplementary Fig. 4c, d), promoted soft agar colony formation (Fig. 4e), and enhanced trans-well migration (Fig. 4f). These results indicate that KLF5 overexpression promotes oncogenic phenotypes in AR-positive prostate cancer cells.

### KLF5 and AR interact on chromatin and regulate opposing transcriptional programs

**Fig. 5:**
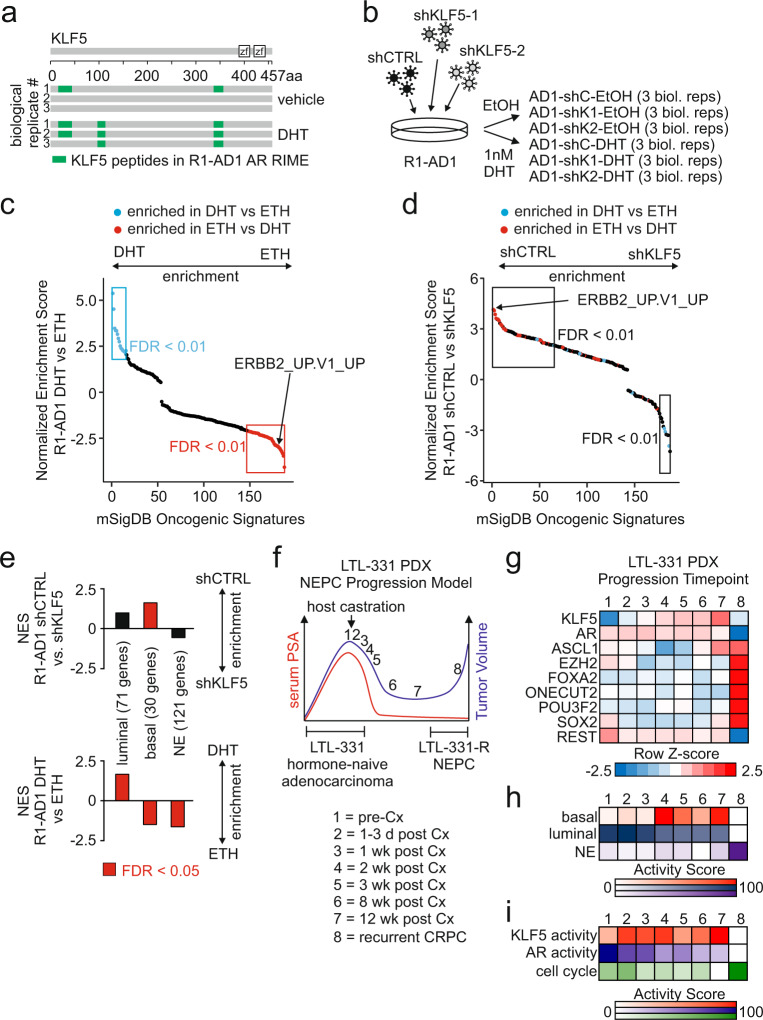
KLF5 and AR display opposing regulation of oncogenic gene sets and genes defining basal cell identity. **a** KLF5 peptides (green) identified in biological replicate AR-targeted RIME experiments using R1-AD1 cells cultured with 1 nM DHT or vehicle (0.1% ethanol) control. **b** RNA-seq experimental design for knockdown of KLF5 with two independent shRNAs targeting KLF5 (shKLF5-1 and −2) or control shRNA (shCTRL), cultured in an androgen-deplete medium, and treatment with 1 nM DHT or vehicle control (0.1% v/v ethanol, ETH) for 24 h. **c** Normalized enrichment scores for all 189 MSigDB oncogenic signatures derived from gene set enrichment analysis (GSEA) using R1-AD1 gene expression data reflecting AR activity (differential expression under DHT vs. ETH). **d** GSEA-derived normalized enrichment scores as in **c** using R1-AD1 gene expression data reflecting KLF5 activity (differential expression in shCTRL vs. shKLF5). Oncogenic Signatures are colored blue or red based on whether they were positively or negatively enriched in **c** with FDR < 0.01. **e** GSEA-deriv**e**d normalized enrichment scores (NES) of luminal, basal, or neuroendocrine (NE) cell gene sets using R1-AD1 gene expression data reflecting KLF5 activity (differential expression in shCTRL vs. shKLF5, top) or AR activity (differential expression in DHT vs. ETH, bottom). **f** Schematic of longitudinal gene expression time-points during adenocarcinoma to neuroendocrine CRPC (NEPC) progression of LTL-331 patient-derived xenografts (PDX). **g** Z-scores of individual gene expression at time-points as in **f**. **h** Activity scores derived from the expression of gene sets distinguishing prostate epithelial cell types (same gene sets as **e**) assessed at time-points as in **f**. **i** Activity scores derived from the expression of gene sets reflecting KLF5 activity, AR activity, and cell cycle progression assessed at time-points as in **f**.

Using rapid immunoprecipitation and mass spectrometry of endogenous proteins (RIME) with a validated AR antibody in DHT-treated R1-AD1 cells, we identified KLF5 peptides in AR immunoprecipitates (Fig. 5a). We used co-immunoprecipitation in R1-AD1 cells to confirm an interaction between FLAG-tagged KLF5 and endogenous AR (Supplementary Fig. 5). To study the transcriptional consequences of KLF5 overexpression and interaction with AR, we performed RNA sequencing (RNA-seq) to identify KLF5 and AR target genes in R1-AD1 cells (Fig. 5b). We confirmed KLF5 transcriptional output in R1-AD1 cells was relevant to CRPC by identifying genes that were positively or negatively correlated with KLF5 expression in CRPC metastases^30,31^, and showing these genes were positively or negatively enriched in R1-AD1 RNA-seq data as a function of KLF5 expression (Supplementary Fig. 6a, b and Supplementary Data 1).

Next, we identified genes that displayed differential regulation by KLF5 and/or ligand-activated AR (Supplementary Data 2–5). KLF5 up-regulated genes outnumbered KLF5 down-regulated genes, indicating that KLF5 was functioning mainly as a transcriptional activator (Supplementary Fig. 7a, b). In R1-AD1 cells with KLF5 knocked down, there was a 2-fold increase in the number of DHT-responsive genes relative to control cells, suggesting that KLF5 was suppressing AR transcriptional output (Supplementary Fig. 7c, d). However, KLF5 knockdown did not affect a set of canonical AR target genes involved in luminal cell homeostasis (Supplementary Fig. 7e).

To probe the interplay between KLF5 and AR, we tested oncogenic gene sets in the molecular signatures database (MSigDB)^32^ for associations with KLF5 or AR activity using gene set enrichment analysis (GSEA)^33^. Strikingly, many of the MSigDB oncogenic signatures that were negatively correlated with active AR (that is, enriched in vehicle vs. DHT-treated cells, depicted in red in Fig. 5c) were positively correlated with active KLF5 (that is, enriched in the shCTRL vs. shKLF5 cells, depicted in red in Fig. 5d). One of these gene sets was ERBB2_UP.V1_UP, which represents a set of 190 genes upregulated in cells expressing the constitutively active c-erbB-2 (ERBB2 or HER2/neu) oncoprotein^34^. Overall, oncogenic gene sets that displayed positive correlation with active AR were more likely to display negative correlation with active KLF5 (depicted in blue in Fig. 5c, d), and gene sets that displayed positive correlation with active KLF5 were more likely to display negative correlation with active AR (depicted in blue in Supplementary Fig. 8a, b, *P* = 0.00047, Fisher’s exact test shown in Supplementary Fig. 8c). These data demonstrate that AR and KLF5 have opposing regulatory effects on many MSigDB oncogenic gene sets with high relevance to cancer biology.

### KLF5 and AR differentially regulate transcriptional programs of prostate epithelial cell identity

We further evaluated these opposing regulatory effects in the context of KLF5 and AR being expressed in different epithelial cell lineages of benign prostate tissue (basal and luminal). Using GSEA to test gene sets that distinguish the three main prostate epithelial cell lineages^35^ revealed that a 30 gene basal cell identity signature was positively enriched in R1-AD1 RNA-seq data reflecting active KLF5, but negatively enriched in R1-AD1 RNA-seq data reflecting active AR (Fig. 5e, Supplementary Fig. 8d). As expected, a 121 gene neuroendocrine cell identity signature was also negatively enriched in RNA-seq data reflecting active AR and a 71 gene luminal cell identity signature was positively enriched in RNA-seq data reflecting active AR (Fig. 5e, Supplementary Fig. 8d). These data indicate that KLF5 activates genes defining basal cell identity, while active AR represses these basal genes and activates genes defining luminal cell identity.

An emerging clinical problem arising from the increased use of potent AR-targeted therapies is prostate cancer lineage plasticity associated with NEPC and reduced/lost dependence on AR^36^. In RNA-seq data from metastatic CRPC biopsies^37^, KLF5 mRNA levels were negatively correlated with an AR activity score and positively correlated with an NEPC score (Supplementary Fig. 9a, b)^38^. CRPC PDXs and a CRPC liver biopsy characterized as NEPC displayed intense staining for KLF5 (Supplementary Fig. 10a, b). Additionally, genes that were positively-regulated by KLF5 in R1-AD1 cells were positively enriched in gene expression data comparing clinical NEPC vs. CRPC tissues (Supplementary Fig. 10c, d) as well as gene expression data comparing NEPC vs. primary prostate cancer tissues (Supplementary Fig. 10e, f). However, active KLF5 did not appear to regulate genes defining neuroendocrine cell identity (Fig. 5e).

To evaluate whether opposing activities of KLF5 and AR were associated with temporal evolution of CRPC to NEPC, we studied gene expression data from the LTL-331 PDX tumor model of castration-induced progression from adenocarcinoma to NEPC (Fig. 5f)^39^. Pre-castrate LTL-331 tumors displayed low KLF5 expression, high AR expression (Fig. 5g), and a high luminal activity score (Fig. 5h). Castration-relapsed NEPC LTL-331-R tumors displayed low AR expression, high expression of the NEPC transcriptional drivers ASCL1, EZH2, FOXA2, ONECUT2, POU3F2/BRN2, and SOX2, low expression of the neuronal repressor REST (Fig. 5g), and a high neuroendocrine activity score (Fig. 5h)^36^. The interval spanning this transition was marked by KLF5 up-regulation (Fig. 5g), the emergence of a high basal activity score, and erosion of luminal activity score (Fig. 5h). We also derived AR and KLF5 activity scores using 19 AR target genes and 37 KLF5 target genes. We found that KLF5 activity was highest during the interval where KLF5 expression and basal activity were high, whereas AR activity declined during this interval (Fig. 5i). Cell cycle activity scores were highest in NEPC LTL-331-R tumors but lowest when KLF5 expression and basal activity scores were high (Fig. 5i). Collectively, these results support a role for KLF5 in opposing AR to promote features of basal cell identity, which precedes the emergence of neuroendocrine hallmarks and castration-resistant tumor growth in a model of NEPC progression.

### *ERBB2* and *ERBB2*-related genes are direct targets of KLF5 and AR opposition

**Fig. 6:**
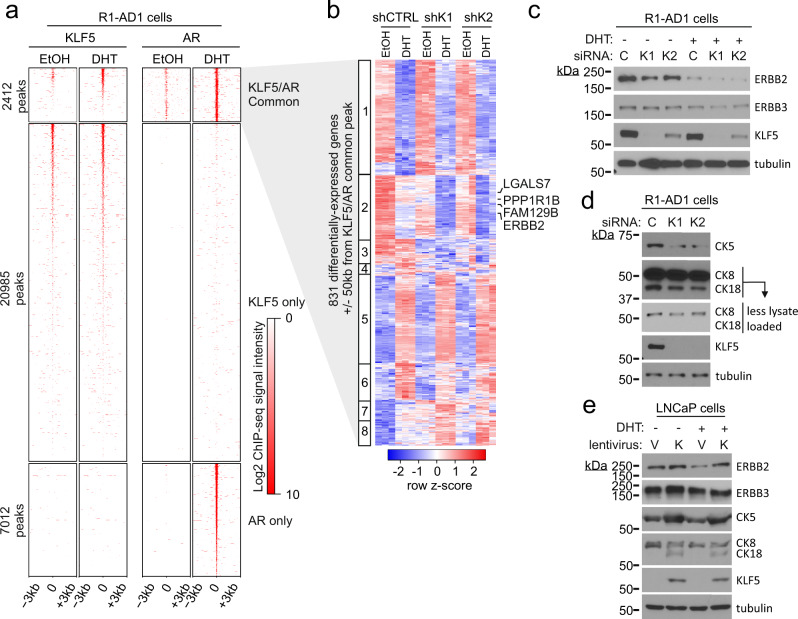
Transcriptional opposition of AR and KLF5 converges at ErbB pathway genes and basal-luminal cell markers. **a** Heatmaps of AR and KLF5 ChIP-seq signals ±3 kb around KLF5/AR common, KLF5 only, or AR only peaks in R1-AD1 cells cultured in an androgen-deplete medium treated 4 h with 1 nM DHT or vehicle control (ethanol, EtOH). **b** Heatmap of RNA-seq gene expression data for a set of 831 genes located ±50 kb from a KLF5/AR common peak and differentially expressed in R1-AD1 cells based on comparisons between shRNA control (shCTRL) vs. shKLF5 conditions and/or DHT vs. vehicle conditions. The heatmap was generated by unsupervised clustering, revealing eight main clusters. **c** Proteins measured by western blot in R1-AD1 cells transfected with KLF5-targeted siRNAs (siK1 and siK2) or control siRNA (siC) were cultured in androgen-deplete medium supplemented with 1 nM DHT or vehicle control (EtOH). Tubulin is a loading control. One additional replicate experiment was performed that yielded a comparable result. **d** Cytokeratin-5 (CK5), cytokeratin 8/18 (CK8/18), and KLF5 proteins measured by western blot in R1-AD1 cells transfected with siRNAs as in **c**. Tubulin is a loading control. One additional replicate experiment was performed that yielded a comparable result. **e** Proteins measured by western blot in LNCaP cells infected with lentivirus encoding KLF5 or empty vector and cultured in an androgen-deplete medium supplemented with 1 nM DHT or vehicle control (EtOH). Tubulin is a loading control. One additional replicate experiment was performed that yielded a comparable result.

To identify direct transcriptional targets of KLF5, we performed KLF5 ChIP-seq in R1-AD1 cells treated with DHT or vehicle control. We identified 23,397 peaks that represented KLF5 binding sites under either of these two conditions. Integrating KLF5 ChIP-seq and AR ChIP-seq data showed that 20,985 binding sites were uniquely engaged by KLF5 (KLF5 only sites), 7,012 binding sites were uniquely engaged by AR (AR only sites), and 2,412 binding sites were engaged by both AR and KLF5 (KLF5/AR common sites) (Fig. 6a). AR and KLF5 motifs were the top motifs enriched at KLF5/AR common sites (Supplementary Fig. 11), indicating motif proximity as the molecular basis for AR/KLF5 interaction.

We integrated RNA-seq and ChIP-seq data to identify 831 genes regulated by KLF5 or DHT and located within 50 kb of a KLF5/AR common binding site. Gene ontology analysis of these 831 genes revealed enrichment of the epidermal growth factor receptor (EGFR) pathway (Supplementary Fig. 12). Unsupervised clustering of these 831 genes revealed eight main patterns of the regulation (Fig. 6b). Genes in Cluster 2 displayed repression by DHT and activation by KLF5 and contained *ERBB2*, *LGALS7* (encodes galectin 7, which regulates activity of ERBB2^40^), *PPP1R1B* (encodes protein phosphatase 1 regulatory inhibitor subunit 1B, which is associated with resistance to the ERBB2-targeted antibody therapeutic trastuzumab^41^), and *FAM129B* (encodes a Ras-binding protein that can be phosphorylated by EGFR to activate Ras signaling^42^).

We validated KLF5-mediated regulation of these targets in R1-AD1 cells using RT-PCR (Supplementary Fig. 13a). ERBB2 protein levels in R1-AD1 cells were highest under androgen-depleted conditions with KLF5 expressed and lowest under androgen-replete conditions with KLF5 knocked down (Fig. 6c). The EGFR family member ERBB3 was insensitive to KLF5 knockdown (Fig. 6c). Knockdown of KLF5 also reduced expression of the basal cell marker CK5, as well as the luminal marker CK18, but not the luminal marker CK8 (Fig. 6d).

Overexpression of KLF5 in LNCaP cells up-regulated ERBB2, CK5, and CK18 proteins, and down-regulated CK8 protein (Fig. 6e). Knockdown of KLF5 in LNCaP sub-lines 16D and 42D down-regulated CK5 protein and up-regulated CK8 protein, with the magnitude being highest in CRPC 16D cells (Supplementary Fig. 13b, c). Knockdown of KLF5 in PC-3 cells down-regulated *LGALS7* and *FAM129B*, but *ERBB2* and *PPP1R1B* were insensitive (Supplementary Fig. 13d). Collectively, these data demonstrate that KLF5 overexpression regulates *ERBB2* expression in AR-positive prostate cancer cells and more broadly regulates ERBB2-related genes *LGALS7* and *FAM129B* in prostate cancer cells regardless of cellular AR status. Consistently, KLF5 promoted the expression of the basal cytokeratin CK5 in AR-positive prostate cancer cells, which supports a role for KLF5 in lineage plasticity.

### KLF5 maintains *ERBB2* expression in a model of enzalutamide-resistant CRPC

**Fig. 7:**
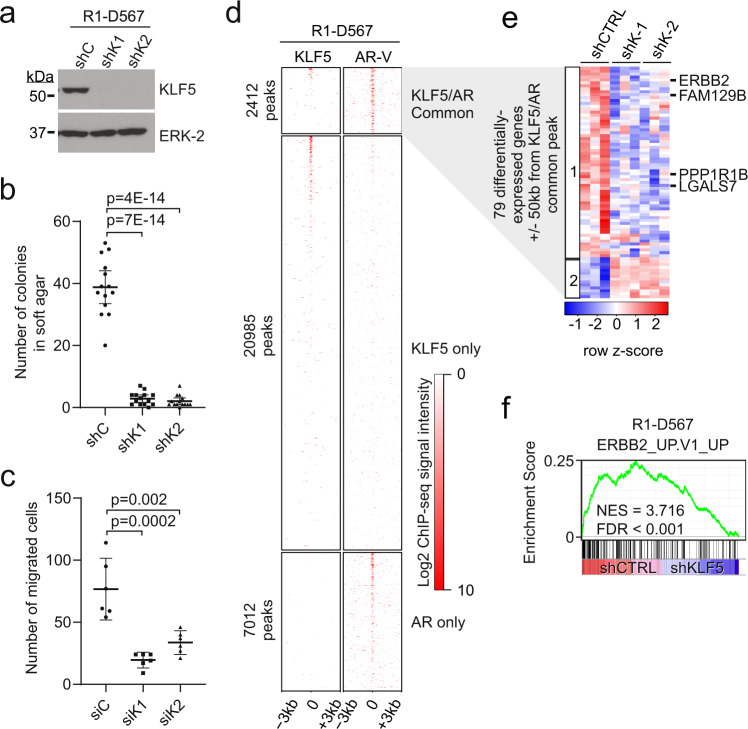
KLF5 retains oncogenic function and activates ERBB2 in an enzalutamide-resistant CRPC cell line model of AR-V activity. **a** KLF5 protein measured by western blot using R1-D567 cells infected with control shRNA lentivirus (shC) or two independent KLF5-targeted shRNA lentiviruses (shK1 and shK2). ERK2 is a loading control. **b** R1-D567 cells as in **a** analyzed by 3D soft agar colony formation assays. *n* = 14, mean ± 95% CI from four independent experiments, two performed in biological triplicate and two performed in biological quadruplicate. *P*-values are unadjusted from 2-sided, 2-tailed *t*-tests. **c** R1-D567 cells transfected with KLF5-targeted siRNAs (siK1 and siK2) or control siRNA (siC) analyzed by chemotactic migration assays. *n* = 6, mean ±95% CI, two independent experiments in biological triplicate. *P*-values are unadjusted from 2-sided, 2-tailed *t*-tests. **d** Heatmaps of AR variant (AR-V) and KLF5 ChIP-seq signals ±3 kb around KLF5/AR common, KLF5 only, or AR only peaks in R1-D567 cells cultured in androgen-deplete medium. **e** Heatmap of RNA-seq gene expression data for a set of 79 genes located ±50 kb from a KLF5/AR common peak and differentially expressed in R1-D567 cells based on comparisons between shRNA control (shCTRL) vs. shKLF5 conditions and/or DHT vs. vehicle conditions. The heatmap was generated by unsupervised clustering, revealing two main clusters. **f** GSEA testing enrichment of the signature ERBB2_UP.V1_UP in R1-D567 gene expression data reflecting active KLF5 (differential expression in shCTRL vs. shKLF5).

In soft agar assays, the growth of AR-positive prostate cancer cells overexpressing KLF5 was suppressed by androgen withdrawal or enzalutamide treatment (Fig. 4b, e), and cell cycle activity was lowest in LTL-331 tumors when KLF5 expression and basal cell activity were highest (Fig. 5g–i). This indicated that KLF5 overexpression alone is insufficient to drive castration- or enzalutamide-resistant cell proliferation but may enable or cooperate with subsequent events that drive this phenotype. Therefore, we tested the effects of KLF5 overexpression in R1-D567 cells, which contain a genetic deletion of *AR* exons 5–7 and display enzalutamide-resistant growth supported by the truncated, constitutively active AR variant protein, ARv567es^43^. In RIME experiments with R1-D567 cells, KLF5 peptides were observed in ARv567es pull-downs (Supplementary Fig. 14a). Further, FLAG-tagged KLF5 interacted with endogenous ARv567es in R1-D567 cells (Supplementary Fig. 14b). Knockdown of KLF5 in R1-D567 cells reduced soft agar colony formation and trans-well migration phenotypes (Fig. 7a–c), and modestly reduced cellular growth in 2D assays (Supplementary Fig. 15).

In ChIP-seq experiments, KLF5/AR common sites displayed co-localization of KLF5 and ARv567es (Fig. 7d). We also used RNA-seq to identify KLF5-regulated genes in R1-D567 cells (Supplementary Fig. 16a–d, Supplementary Data 6). In R1-D567 cells, KLF5 maintained positive regulation of *ERBB2*, *LGALS7*, *PPP1R1B*, and *FAM129B* expression (Fig. 7e, Supplementary Fig. 17) as well as the broader ERBB2_UP.V1_UP gene signature (Fig. 7f). These data demonstrate that KLF5 maintains oncogenic activities and activates *ERBB2* and related genes in a context where cooperative events, such as expression of an AR variant, drive enzalutamide resistance.

### Therapeutic targeting of ERBB2 blocks oncogenic KLF5 activities

**Fig. 8:**
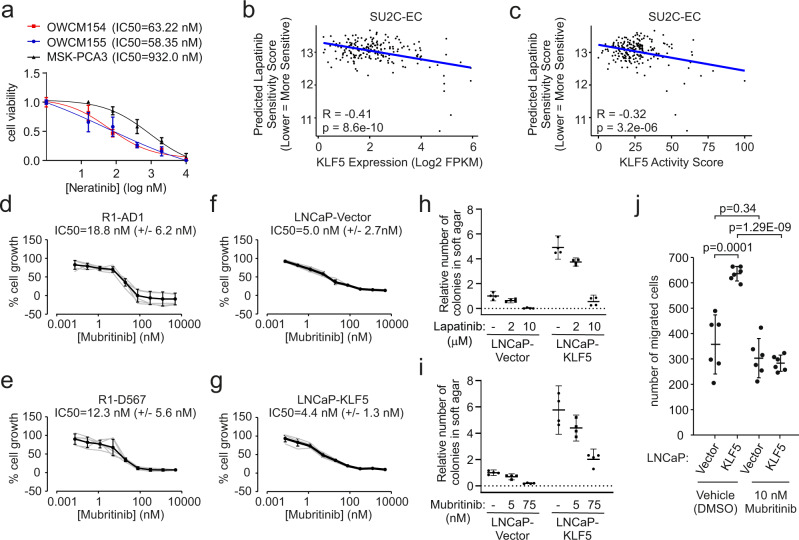
Targeting ERBB2 inhibits oncogenic effects of KLF5. **a** Viability assays of patient-derived organoids developed from neuroendocrine CRPC (OWCM154 and 155) or CRPC adenocarcinoma (MSK-PCA3) treated with neratinib. Data are mean ±95% CI from three independent experiments in biological triplicate (*n* = 9). **b**, Lapatinib sensitivity scores predicted using ridge regression models trained on high-throughput cancer cell line drug screens in metastatic CRPC tumors (SU2C-EC study), plotted vs. KLF5 expression (FPKM = fragments per kb per million fragments mapped). Pearson correlation coefficients (R) and 2-tailed *p*-values for r ≠ 0 are shown. **c** Lapatinib sensitivity scores as in **b** plotted vs. KLF5 activity score. **d–g** 2-dimensional growth assays for R1-AD1 cells, R1-D567 cells, LNCaP cells infected with empty lentivirus (LNCaP-Vector), and LNCaP cells infected with lentivirus encoding KLF5 (LNCaP-KLF5) cultured in androgen-replete medium and treated for 5 days with mubritinib. Gray lines are biological replicates (*n* = 6), black lines are mean ±95% CI, IC50 values are mean ±95% CI. **h, i** LNCaP cells as in **f** and **g** were analyzed by 3D soft agar colony formation assays in androgen-replete medium supplemented with lapatinib or mubritinib. *n* = 4, mean ±95% CI, two independent experiments in biological duplicate. **j** LNCaP cells as in **f** and **g** analyzed by chemotactic migration assays with the medium in the top chamber containing 10 nM mubritinib or vehicle control (DMSO). *n* = 6, mean ±95% CI, two independent experiments in biological triplicate. *P*-values are from unpaired 2-sided *t*-tests.

In the clinic, the dual EGFR/ERBB2 inhibitors lapatinib and neratinib are used as therapies for ERBB2/HER2-positive breast cancer. A previous drug screen with NEPC patient-derived organoids (PDOs) identified sensitivity to neratinib plus an EZH2 inhibitor^44^. We found that neratinib as a single agent displayed IC_50_ values that were 14–16-fold lower in NEPC PDOs OWCM154 and OWCM155 when compared with a CRPC adenocarcinoma PDO MSK-PCA3 (Fig. 8a). OWCM154 and OWCM155 also displayed higher average expression of 37 genes reflecting KLF5 activity compared to MSK-PCA3 (Supplementary Fig. 18a, b) even though KLF5 expression was higher in OWCM154 and lower in OWCM155 compared to MSK-PCA3 (Supplementary Fig. 18c). Next, we used a computational method for predicting drug responses in patients based on their baseline tumor gene expression profiles^45^, using RNA-seq data from metastatic CRPC^37^ and primary tumors^46^. Higher KLF5 expression was associated with scores that predicted greater lapatinib sensitivity in both datasets (Fig. 8b and Supplementary Fig. 18d). Higher KLF5 activity scores predicted greater lapatinib sensitivity in the dataset derived from metastatic CRPC but not the dataset derived from primary tumors (Fig. 8c and Supplementary Fig. 18e).

We tested lapatinib in the KLF5-high R1-AD1 and R1-D567 models, and observed IC_50_ values in the low-μM range for growth inhibition (Supplementary Fig. 19). Because lapatinib and neratinib are inhibitors of EGFR1 and ERBB2, we also tested the ERBB2-selective inhibitor mubritinib. In R1-AD1 and R1-D567 cells, IC_50_ values for growth inhibition were in the low-nM range (Fig. 8d, e). The IC_50_ value for mubritinib in KLF5-low LNCaP cells was also in this low-nM range but did not change with KLF5 overexpression (Fig. 8f, g). Across these 2D growth assays in cell line models, KLF5 levels did not correlate with sensitivity to ERBB2 inhibitors.

We next considered our findings that KLF5 affected phenotypes of migration and anchorage-independent growth in soft agar, but had minimal effects in 2D-growth assays. If ERBB2 is an important effector downstream of KLF5, the effects of ERBB2 inhibition may not manifest in 2D-growth assays. Therefore, we tested the effects of ERBB2 inhibitors in soft agar colony formation assays and trans-well migration assays. Lapatinib and mubritinib inhibited the increase in soft agar colony formation mediated by KLF5 overexpression in LNCaP cells (Fig. 8h, i). Further, mubritinib blocked the trans-well migration that was driven by KLF5 overexpression in LNCaP cells (Fig. 8j). These results support the concept that ERBB2 activity is regulated by KLF5 in prostate cancer cells. These results also indicate that ERBB2 could represent a therapeutic target to prevent or reverse oncogenic phenotypes driven by KLF5 overexpression during CRPC progression.

## Discussion

In this study, we found that AR-negative basal cells in the normal prostate express high levels of KLF5, whereas AR-positive luminal cells express low KLF5. This reciprocal relationship persists in localized prostate cancer, where KLF5 expression is low/absent and tumor cells have an AR-positive luminal identity. Low/absent KLF5 in localized prostate cancer is consistent with reports of KLF5 being a prostate tumor suppressor^7–9^. Androgen-dependent prostate cancer cell lines LNCaP and VCaP also expressed low levels of KLF5. In contrast, a subset of CRPC tumors, PDXs, and CRPC cell lines displayed KLF5 overexpression.

We identified two distinct mechanisms that up-regulate KLF5 expression in prostate cancer cells. First, androgens transiently up-regulate KLF5 expression, and exposure to AR-targeted therapies increases the durability of this response. For instance, stronger and more durable upregulation of KLF5 by androgens occurred in LNCaP cells after just 3 weeks of exposure to enzalutamide. Second, we found that a *KLF5* super-enhancer active in other cancers displayed a high density of active chromatin marks in PC-3 cells and that KLF5 expression in PC-3 cells was insensitive to AR re-expression. The transcriptional and epigenetic regulators that control this AR-independent mechanism of KLF5 activation remain undefined but are likely linked to the changes in KLF5 permissiveness for transient vs. durable androgen up-regulation in cells treated with antiandrogens. *KLF5* mRNA translation is also negatively regulated by androgens^21^, which together with these transcriptional mechanisms establishes a disease context where AR-targeted therapy can lead to high KLF5 levels in AR-positive CRPC cells.

In this context of KLF5 and AR co-expression, transcriptional programs driven by KLF5 physically and functionally opposed androgen/AR transcriptional programs, including regulation of a gene set defining basal cell identity. This is in disagreement with a prior finding that KLF5 co-operates with AR to activate canonical androgen/AR responsive genes *PSA*, *TMPRSS2*, and *FKBP5*^25^. One potential explanation for this discrepancy could be that this prior study knocked down KLF5 in LNCaP cells, which express negligible levels of KLF5. Additionally, low vs. high KLF5 expression levels may impact the stoichiometry of KLF5 acetylation, which regulates KLF5 protein activity^6^. In our study with models of KLF5 overexpression, there was no evidence for KLF5 affecting the regulation of canonical AR targets that are mainly involved in the homeostasis of prostate luminal cells. Noteworthy, these canonical targets are also unaffected by loss of *TP53* and *RB1*, which also promotes the expression of a basal cell signature and CRPC lineage plasticity^47^.

Our finding of KLF5 and AR opposition provides clues to the molecular determinants of KLF5 functioning as an oncogene vs. tumor suppressor in prostate cancer. In early stages of prostate cancer, tumor cells have luminal identities and their growth and survival is AR-dependent^3^. In this context, KLF5 opposition of AR would be expected to have a tumor-suppressive effect, and could explain why *Klf5* deletion in the mouse prostate accelerates tumorigenesis driven by *Pten* loss^7^. Alternatively, CRPC tumors consist of populations of cells that have adapted to AR inhibition via myriad somatic alterations to the *AR* gene and extensive reprogramming of AR cistromes^15^. This reprogramming may create an environment where KLF5 activity is tolerable and oncogenic effects can manifest. In our study the oncogenic effects of KLF5 were context-specific, promoting migration and colony formation in soft agar but modestly affecting cell proliferation. Consistent with context-specificity, acetylated KLF5 was shown to promote growth, epithelial to mesenchymal transition (EMT), and docetaxel resistance in intra-tibial xenografts of DU145 and PC-3 cells^29^, but inhibit invasion of DU145 and PC-3 cell lines in vitro and in vivo via the IGF1-STAT5 pathway^9^. Further, although our study showed that LNCaP and C4-2B cells contain low levels of KLF5, a separate study that knocked down KLF5 in these cells showed inhibition of androgen-dependent proliferation in vitro and in vivo^9,25^. Collectively, these data point to KLF5 having divergent effects on cancer phenotypes, which are likely influenced by AR levels, epithelial cell identity, KLF5 levels, KLF5 acetylation, and hormonal milieu, among other factors.

Our finding that KLF5 and AR opposition converged on a set of genes that define basal cell identity, and that KLF5 overexpression in AR-positive prostate cancer cells enhanced expression of the basal cytokeratin CK5, highlights a role for KLF5 in supporting a mixed basal/luminal cell identity. In the LTL-331 model of NEPC progression, the emergence of a high basal cell identity score coincided with upregulation of KLF5 and bridged the transition from a luminal cell identity to neuroendocrine cell identity. Future studies are warranted to test whether this mixed basal/luminal identity promoted by KLF5 is a common, early step in CRPC lineage plasticity that enables subsequent reprogramming to establish NEPC or other cell lineage states observed in advanced CRPC^36^.

Prior studies found that ERBB2 enhances AR mRNA degradation, protein stability, and transcriptional activity^48–50^. Our work adds to these ERBB2/AR regulatory relationships by establishing ERBB2 as a point of convergence of AR and KLF5 transcriptional activities. We found that ERBB2 inhibitors blocked oncogenic phenotypes promoted by KLF5, including migration and colony formation in soft agar. Conversely, potencies of ERBB2 inhibitors in cell proliferation assays were insensitive to KLF5 levels, which is noteworthy because cell proliferation regulation by KLF5 in 2D assays was negligible. In a Phase II trial, lapatinib displayed clinical activity in 2/29 CRPC patients^51^, suggesting a potential for efficacy in specific contexts. Our computational model suggested that KLF5 expression or activity might serve as biomarkers of higher lapatinib sensitivity in CRPC. Further, NEPC PDO viability was more sensitive to neratinib than the viability of a PDO representing CRPC adenocarcinoma. Interestingly, combination therapy of LNCaP xenografts with lapatinib plus enzalutamide reduced growth more effectively than either therapy alone^52^. Our studies indicate the potential for this combination to prevent or delay the progression of CRPC to more advanced and lethal phenotypes.

## Methods

### Cell lines

LNCaP, VCaP, 22Rv1, DU145, PC-3, and NCI-H660 cells were obtained from American Type Culture Collection (ATCC). R1-AD1 and R1-D567 cells have been previously described^43^. LNCaP sub-lines derived from xenografts in castrated or castrated/enzalutamide-treated mice (V16D, 49F, and 42D) have been described^16^. LNCaP95 cells were a gift from Jun Luo (Johns Hopkins University). C4-2B cells were a gift from Leland Chung (Cedars Sinai). R1-AD1, LNCaP (parental and V16D, 49F, and 42D), 22Rv1, DU145, and PC-3 cells were maintained in Roswell Park Memorial Institute (RPMI) 1640 medium (Gibco) with 10% fetal bovine serum (FBS) and antibiotics (penicillin and streptomycin). Medium for 49F and 42D was supplemented with 10 μM enzalutamide (MedChem Express). R1-D567 and LNCaP95 were cultured in RPMI 1640 medium with 10% charcoal-stripped FBS (CSS). VCaP cells were cultured in Dulbecco’s modified Eagle’s medium (DMEM) plus 10% FBS and antibiotics. NCI-H660 cells were maintained according to ATCC protocol with supplements. For androgen depletion and repletion experiments, cells were seeded in corresponding media containing 10% CSS for 48 h and stimulated for indicated time-points with medium containing 0.1–10 nM dihydrotestosterone (DHT, Sigma), 0.1–10 nM R1881 (Sigma-Aldrich), or 1 nM mibolerone (Biomol). Cell line authentication and mycoplasma testing procedures are described in the “Supplementary Materials and Methods”.

### Antibodies and siRNA reagents

siRNAs targeted to KLF5, siKLF5-1 (siK1, 5′-CGAUUACCCUGGUUGCACA-3′) and siKLF5-2 (siK2, 5′-AAGCUCACCUGAGGACUCA-3′), were purchased from Dharmacon.

Antibodies were purchased from Santa Cruz for western blot detection of AR (N-20, sc-816, 1:1000), tubulin (B-5-1-2, sc-23948, 1:3000), ERK2 (D-2, sc-1647, 1:4000), and KLF5 (Santa Cruz, G7, sc-398470, 1:2000). Antibodies were purchased from Cell Signaling for western blot detection of ERBB2 (29D8, #2165, 1:1000) and ERBB3 (D22C5, #12708, 1:1000), Cell Marque for western blot detection of AR (SP107, 200R-16, 1:4000), Sigma for western blot detection of CK5 (SAB4501651, 1:1000) and from Leica for western blot detection of CK8/18 (NCL-L-5D3, 1:3000).

### Rapid immunoprecipitation and mass spectrometry of endogenous proteins (RIME)

RIME with R1-AD1 and R1-D567 cells has been described^53^.

### Western blot

Cells were lysed in 1X Laemmli buffer (65 mM Tris-HCl, pH7.0, 2% (w/v) SDS, 5% (v/v) β-mercaptoethanol, 10% (v/v) glycerol, and 0.5% (w/v) bromophenol blue) and homogenized using a 28-gauge insulin syringe (BD). Equal protein masses of lysates were separated by electrophoresis in 7.5% PAGE gels, followed by transfer to PVDF membranes (Immobilon-P, Millipore). Membranes were incubated with primary antibodies overnight at 4 °C and the HRP-conjugated secondary antibodies at room temperature for 1 h. Blots were incubated with Super Signal West Pico (Thermo) and exposed to X-ray film or imaged using an iBright CL750 system (Thermo Fisher).

### RT-PCR

Total RNA was extracted from cells using the ReliaPrep RNA miniprep systems (Promega) according to manufacturer instructions. Total RNA (1 μg) was reverse transcribed using the GoScript Reverse Transcription Kit (Promega) and an oligo (dT) primer according to manufacturer instructions. cDNA from *Pten*−/− mouse organoids have been described^21^. Quantitative PCR was performed on a Bio-Rad CFX Connect Real-Time Detection System using SYBR Green Master Mix (Bio-Rad) and 1 μL of cDNA as input in a 20 μL reaction with primers listed in Supplementary Table 1 or purchased from Applied Biosystems (GAPDH). Cycle thresholds (Ct) were determined using Bio-Rad CFX manager software. Relative quantification was used to determine fold change in expression levels by comparative Ct method using the formula 2^ −ΔΔCt^ with *GAPDH* or *ACTB* as calibrator. PCR reactions were performed in technical duplicate or triplicate, with at least two biological replicates (*n* ≥ 6).

### ChIP-seq analysis of KLF5, AR, and H3K27ac in prostate cancer cell lines

Chromatin immunoprecipitation (ChIP) with an antibody specific for KLF5 (H300, Santa Cruz) was performed as two independent biological replicate experiments as described previously^54^ with the following modifications: R1-AD1 and R1-D567 cells were seeded at 5 × 10^6^ cells/plate on 100 mm dishes in RPMI 1640 containing 10% CSS, allowed to adhere for 72 h, then re-fed for another 4 h with RPMI 1640 containing 5% CSS with ethanol (0.1% v/v) or 1 nM DHT prior to fixation. Nuclear extracts were subjected to sonication on dry ice for eight cycles at 40% amplitude using a 450 Sonifier (Branson). Each cycle consisted of 10 s pulse / 10 s rests for 1 min, with 5 min rests between cycles to achieve DNA fragmentation of 200–500 bp. Lysates were pre-cleared by 1 h incubation with Protein A/G Plus agarose beads (GE) pre-blocked with tRNA (Sigma). Lysates were immunoprecipitated overnight with 5 μg KLF5 antibody and tRNA-blocked Protein A/G Plus agarose beads. DNA was purified using a PCR purification kit (Qiagen), and 2 ng of input or ChIP-enriched DNA was submitted to the University of Minnesota Genomics Center for library preparation with a Rubicon ChIP Sample Preparation Kit (Illumina) and sequencing using an Illumina HiSeq 2500 at 1 × 50 bp settings in high output mode. ChIP-seq with antibodies specific for AR in R1-AD1 and AR-V in R1-D567 cells has been described^54^ and data are available in NCBI Gene Expression Omnibus (GEO accession GSE61838). H3K27ac ChIP-seq data from LNCaP, VCaP, and PC-3 cells maintained continuously in androgen-containing medium were obtained from NCBI GEO (LNCaP, GSM3138725; VCaP, GSM2827606; PC-3, GSM1383871). H3K4me1 ChIP-seq data from LNCaP, VCaP, and PC-3 cells maintained continuously in the androgen-containing medium were obtained as .bigwig files from the Cistrome Data Browser^55^, which analyzed raw data from NCBI GEO (LNCaP, GSM353634; VCaP, GSM353631) or the ENCODE project (PC-3, ENCSR566UMF_1). AR ChIP-seq data from LNCaP and VCaP cells treated 4 h with the synthetic androgen R1881 was obtained from NCBI GEO (LNCaP, GSM2480801, and GSM2480803; VCaP, GSM2235688, and GSM2235689).

### ChIP-seq analysis of H3K27ac in clinical CRPC tissues

Metastatic samples from CRPC patients were collected at the Netherlands Cancer Institute-Antoni van Leeuwenhoek, Amsterdam, Netherlands. This study was approved by the institutional METC and IRB and conducted according to the principles of the Declaration of Helsinki (Oktober 2013 version) and in accordance with the Medical Research Involving Human Subjects Act (WMO) and other guidelines, regulations, and Acts. The patient’s materials were handled in accordance with the ‘Code of conduct for responsible use’ (version 2011) as made by the Dutch Federation of Biomedical Scientific Societies. Informed consent was obtained from the participants included in the study. Fresh frozen metastasis samples from CRPC patients were processed using the ChIP-seq protocol as described^56^. ChIP-seq for H3K27ac (Active Motif, Cat#39133) was performed using 5 μg of antibody and 50 μL of Protein A magnetic beads (Invitrogen) per sample. Immunoprecipitated DNA was processed for sequencing using standard protocols and sequenced on an Illumina Hi-seq 2500 with 65 bp single end reads.

### ChIP-seq data analysis

KLF5, AR, and H3K27ac ChIP-seq data sets were mapped using bwa (v. 0.7.17 for cell line data and v. 0.5.10 for tissue data)^57^ against the human (hg19) reference genome. Mapped data were filtered to keep only reads with a mapq = 1. Bam files were filtered using bedtools (v. 2.27.1)^58^ to remove any ENCODE blacklisted regions (ENCFF001TDO.bed). Macs2 (v. 2.1.1)^59^ was used for peak calling using paired treatment and control bam files. Data from treatment replicates were merged to create bam files and bigwigs for visualization. We used the bedtools ‘intersect’ and ‘subtract’ functions to obtain final peak sets. H3K4me1 ChIP-seq data sets were obtained as .bigwig files from the Cistrome Data Browser^55^. For the peak sets derived from KLF5 ChIP-Seq, we reduced the number of peaks by finding the intersect across all replicates. For the peak sets derived from AR ChIP-seq, we used the AR ChIP-seq replicate that yielded the most peaks. Read coverage snapshots were generated using IGV (v. 2.8.2 for cell line data and v. 2.3.93 for tissue H3K27). Individual H3K27ac and H3K4me1 window scales were selected using two times of the average maximum H3K27ac or H3K4me1 peaks height of GAPDH, ACTB, and TUBA1A-C regions.

### Immunohistochemistry with clinical tissues

Tissue microarrays (TMAs) (56 case hormone sensitivity array, 40 Case Screening array, 42 LuCaP PDX Models array), were purchased under materials transfer agreements from the Prostate Cancer Biorepository Network (PCBN). A metastatic biopsy (liver) with AR-/NEPC + histology has been described^60^. Unstained tissue sections (4 µm) were de-paraffinized and rehydrated using standard methods. For antigen retrieval, slides were incubated in 6.0 pH buffer (Reveal Decloaking reagent, Biocare Medical, Concord, CA) in a steamer for 30 min at 95–98 °C, followed by a 20 min cooldown period. Subsequent steps were automated using an immunohistochemical staining platform (Intellipath, Biocare). Endogenous peroxidase activity was quenched by slide immersion in 3% hydrogen peroxide solution (Peroxidazed, Biocare) for 10 min followed by TBST rinse. A serum-free blocking solution (Background Sniper, Biocare Medical, Concord, CA) was placed on sections for 10 min. Blocking solution was removed and slides were incubated in primary antibody diluted in 10% blocking solution/90% TBST. A mouse monoclonal antibody recognizing KLF5 (anti-BTEB2 G-7, Santa Cruz Biotechnology sc-398470) was incubated with blocked TMA slides at 1:200 dilution for 60 min at room temperature followed by TBST rinse and detection with Novocastra Novolink Polymer Kit (Leica Microsystems Inc., Buffalo Grove, IL) using the manufacturer’s specifications. All slides then proceeded with TBST rinse and detection with diaminobenzidine (DAB) (Biolegend, Dedham, MA). Slides were incubated for 5 min followed by TBS rinse then counterstained with CAT Hematoxylin (Biocare, Concord, CA) for 5 min. Slides were then dehydrated and coverslipped. TMA spots were scored by a genitourinary pathologist (P.M.) for antibody staining intensity in carcinoma cells, benign acinar luminal cells, or benign basal cells using a scale of 0–3 as described^61^. For tissue samples that were represented by multiple replicate spots on the TMAs, an average of the individual scores for each spot was used. Differences in staining intensity between tissue types were determined using Mann–Whitney *U*-test based on non-normal data distributions in Shapiro–Wilks goodness of fit tests.

### Plasmid constructs

The non-silencing shRNA pGIPZ lentiviral vector for shRNA control (shC), and the pGIPZ shRNA constructs for KLF5 (V2LHS_150120, V3LHS_333122) were purchased from Open Biosystems (Pittsburgh, PA). The lentivirus overexpression vector pLenti-EF1a-PGK-puro harboring KLF5 cDNA, as well as empty vector, have been described^14^ and were kindly provided by Dr. Matthew Meyerson.

### Soft agar colony formation assays

Two agar layers were generated per well of 6- or 12-well plates, with the bottom layer containing 0.7% (w/v) SeaPlaque agarose (Lonza) in tissue culture growth medium, and the top agar layer containing 1 × 10^4^ cells dispersed in 1 mL of 0.35% (w/v) agarose in tissue culture medium. After the top agar layer solidified at room temperature, 1 mL of tissue culture growth medium containing 3 nM DHT (or ethanol as vehicle control), 30 μM enzalutamide, 5 or 75 nM mubritinib, or 2 or 10 μM lapatinib (or DMSO as vehicle control) was added. The plates were incubated in a tissue culture incubator at 37 °C for 21–28 days, with the top 1 mL of growth medium replaced every 7 days. For visualization of colonies at the assay endpoint, the top 1 mL of growth medium was replaced with fresh growth medium containing 100 μg/μL Iodonitrotetrazolium chloride (INT, Sigma I8377). Images of plates were captured and colonies were counted using Image J software.

### Trans-well migration assays

Haptotactic migration assays were performed as previously described^62^ with modifications. Briefly, cells were suspended in RPMI media containing 10% CSS and seeded at 60,000 cells/well (R1-AD1 and R1-D567) or 30,000 cells/well (LNCaP) cells in 8.0 µm pore size cell culture inserts for 24-well plates coated with 20 µg/mL fibronectin in PBS (Sigma F-1141-1MG). For mubritinib experiments, cells were seeded in a medium containing 10 nM mubritinib or 0.1% (v/v) DMSO as vehicle control. Each well of the 24-well plates with the 8.0 µm pore size cell culture inserts contained RPMI medium supplemented with 20% FBS and antibiotics. After scraping off the non-migratory cells from the top chamber, the migrated cells on the bottom surface of the membrane were fixed in 100% methanol for 15 min and stained with 0.2% crystal violet overnight at 4 °C. Images from each cell culture insert were captured using a Nikon Eclipse TS100 microscope, 10X phase objective, NA = 0.25 equipped with a Nikon digital sight camera and NIS Elements D 4.00.12 software. The experiment was done in three inserts per condition per experiment and repeated two times (*n* = 6).

### RNA-seq

For RNA-seq, 1 μg of isolated total RNA from indicated cells were submitted to University of Minnesota Genomics Center. The library was prepared with TruSeq Stranded mRNA kit (Illumina) according to manufacturer’s instructions and was constructed for 2 × 100 paired-end sequencing on an Illumina HiSeq 2000 system.

### Identification of differentially expressed genes in RNA-seq data

Fastq files containing 75 bp paired-end reads were aligned to the hg19 reference genome using HiSat2 (v. 2.1.0)^63^. Subread (v. 1.4.6)^64^ was used to quantify gene expression using the version 87 GRCh37 annotation from Ensembl^65^. Count data were filtered to only keep genes that had a cpm (counts per million) value greater than 1 cpm in at least two samples across all experimental conditions. The likelihood ratio test was used to evaluate differential expression with edgeR (v. 3.20.9)^66,67^. The Benjamini–Hochberg method was used for multiple hypothesis testing correction. An adjusted *p*-value = 0.01 was used as a differential expression significance threshold. We annotated differentially expressed genes that were within a ±50 kb window of the KLF5/AR common peak bed file from our ChIP-seq analysis.

### Gene set enrichment analysis and gene set activity scoring

Ranked gene lists for Gene Set Enrichment Analysis (GSEA) were generated using the following two-step transformation. First, unadjusted *P*-values from the differential expression tests were transformed by −log10, then the −log10 *P*-values for each gene are multiplied by either +1 or −1 depending on the sign of the expression fold change for that gene (positively regulated genes are multiplied by +1 and negatively regulated genes are multiplied by −1). This ranked gene list was used for “Preranked” GSEA analysis using the GSEA java program (v. 3.0)^33^. We used 10,000 permutations for significance testing instead of the default of 1000 to better control for false discoveries. To enable reproducible testing results, we used a seed of 149 instead of the default timestamp. We tested our ranked gene lists against the oncogenic and hallmark MSigDb collections (v. 6.1). We also tested our ranked gene lists against gene sets that are restricted to basal (30 genes), luminal (71 genes), and neuroendocrine prostate epithelial cells in single-cell RNA-seq data^35^. The 560 gene prostate neuroendocrine gene set was truncated at the top 121 genes (FDR cutoff of 1E-48). Activity scores were determined using summative Z-scores of gene sets, which were converted to percent where 0 is the lowest score and 100 is the highest score as described^30^. All gene sets are in Supplementary Data 1. Activity scores were applied to microarray gene expression data from longitudinal collections of pre- and post-castration LTL-331 PDX tumor tissue^39^ and RNA-seq data from PDOs OWCM154 (GEO accession GSM3083468), OWCM155 (GEO accession GSM3083470), and MSK-PCA3 (GEO accession GSM5501250). MSK-PCA3 RNA-seq data in FPKM (fragments per kb per million) were converted to TPM (transcripts per million) by dividing the FPKM value for that gene in that sample by the sum of all FPKM values in that sample and scaling by 1e6.

### Cell growth assays

Cells were seeded at a density of 2 × 10^4^ cells/well on 24-well plates in RPMI 1640 with 10% CSS, allowed to adhere for 24 h, and the medium was replaced with a medium containing 1 nM DHT or 0.1% v/v ethanol as a control. At indicated time-points, cells were fixed and stained with crystal violet as described^68^ and the absorbance was measured at 560 nM. Significance was assessed by unpaired 2-sided *t*-tests.

### Patient-derived organoid viability assays

OWCM154 and OWCM155 organoids^44^ and MSK-PCA3 organoids^69^ have been described. Cell viability assays were performed using a CellTiter-Glo kit (Promega) according to the manufacturer’s protocol. Organoid cells (4000) were seeded in 96 well, collagen Type I-coated microplates and the next day treated with 16 nM–10 μM neratinib (SelleckChem) or DMSO as vehicle control for 6 days. Cell viability was determined by normalizing raw luminescence units to the median values of the negative control (DMSO) on a per plate basis. IC50 (nM) was calculated using GraphPad Prism 8.

### Statistics and reproducibility

Experiments requiring dedicated statistical tests have those tests described in the appropriate section of the Methods. For all other experiments, differences in mean values were deemed significant if no overlap was noted in 95% confidence intervals. For relative quantification RT-PCR experiments, we illustrated mean ±95% confidence intervals for all data derived from technical replicates within biological replicate experiments to show the experimental variability in measuring reference and non-reference samples. The rationale for this approach is that averaging technical replicates to a single value per biological replicate in a relative quantification RT-PCR experiment makes all reference sample values equal 1.0 exactly, with zero error in measurement of the reference sample. This would not appropriately reflect the technical imprecision in measuring gene expression for reference and non-reference samples by relative quantification RT-PCR. For migration and soft agar colony formation assays, unpaired 2-sided *t*-tests were used to calculate *p*-values for differences in means. For experiments showing a representative western blot, at least one additional replicate experiment was performed that yielded a comparable result.

### Reporting Summary

Further information on research design is available in the Nature Research Reporting Summary linked to this article.

## Supplementary information

Supplementary Information

Description of Additional Supplementary Files

Supplementary Data 1

Supplementary Data 2

Supplementary Data 3

Supplementary Data 4

Supplementary Data 5

Supplementary Data 6

Reporting Summary

## Data Availability

The ChIP-seq and RNA-seq data generated in this study have been deposited in the NCBI GEO database under accession code GSE148808. Full western blot images are in Supplementary Figs. 20-21. Public datasets that were used include: R1-AD1 and R1-D567 AR ChIP-seq (GSE61838), LNCaP H3K27ac ChIP-seq (GSM3138725), VCaP H3K27ac ChIP-seq (GSM2827606), PC-3 H3K27ac ChIP-seq (GSM1383871), LNCaP H3K4me1 ChIP-seq (GSM353634), VCaP H3K4me1 ChIP-seq (GSM353631), PC-3 H3K4me1 ChIP-seq (ENCSR566UMF_1), LNCaP AR ChIP-seq (GSM2480801 and GSM2480803), VCaP AR ChIP-seq (GSM2235688 and GSM2235689). OWCM154 RNA-seq (GSM3083468), OWCM155 RNA-seq (GSM3083470), MSK-PCA3 RNA-seq (GSM5501250). The remaining data are available within the Article and Supplementary Information.

## References

[1] Cunha GR (2018). Development of the human prostate. Differentiation.

[2] Matusik RJ (2008). Prostate epithelial cell fate. Differentiation.

[3] Strand DW, Goldstein AS (2015). The many ways to make a luminal cell and a prostate cancer cell. Endocr. Relat. Cancer.

[4] Ryan CJ, Tindall DJ (2011). Androgen receptor rediscovered: the new biology and targeting the androgen receptor therapeutically. J. Clin. Oncol..

[5] Jiang J (2008). A core Klf circuitry regulates self-renewal of embryonic stem cells. Nat. Cell Biol..

[6] Zhang B (2020). Klf5 acetylation regulates luminal differentiation of basal progenitors in prostate development and regeneration. Nat. Commun..

[7] Xing C (2014). Klf5 deletion promotes Pten deletion-initiated luminal-type mouse prostate tumors through multiple oncogenic signaling pathways. Neoplasia.

[8] Chen C, Bhalala HV, Vessella RL, Dong JT (2003). KLF5 is frequently deleted and down-regulated but rarely mutated in prostate cancer. Prostate.

[9] Ma JB (2020). KLF5 inhibits STAT3 activity and tumor metastasis in prostate cancer by suppressing IGF1 transcription cooperatively with HDAC1. Cell Death Dis..

[10] Chen C (2006). KLF5 promotes cell proliferation and tumorigenesis through gene regulation and the TSU-Pr1 human bladder cancer cell line. Int J. Cancer.

[11] Chia NY (2015). Regulatory crosstalk between lineage-survival oncogenes KLF5, GATA4 and GATA6 cooperatively promotes gastric cancer development. Gut.

[12] Nandan MO (2008). Kruppel-like factor 5 mediates cellular transformation during oncogenic KRAS-induced intestinal tumorigenesis. Gastroenterology.

[13] Tong D (2006). Expression of KLF5 is a prognostic factor for disease-free survival and overall survival in patients with breast cancer. Clin. Cancer Res.

[14] Zhang X (2018). Somatic superenhancer duplications and hotspot mutations lead to oncogenic activation of the KLF5 transcription factor. Cancer Discov..

[15] Watson PA, Arora VK, Sawyers CL (2015). Emerging mechanisms of resistance to androgen receptor inhibitors in prostate cancer. Nat. Rev. Cancer.

[16] Bishop, J. L. et al. The master neural transcription factor BRN2 is an androgen receptor suppressed driver of neuroendocrine differentiation in prostate cancer. *Cancer Discov.* 7, 54–71 (2016). 10.1158/2159-8290.CD-15-1263 27784708

[17] Dardenne E (2016). N-Myc induces an EZH2-mediated transcriptional program driving neuroendocrine prostate cancer. Cancer Cell.

[18] Guo H (2019). ONECUT2 is a driver of neuroendocrine prostate cancer. Nat. Commun..

[19] Shukla S (2017). Aberrant activation of a gastrointestinal transcriptional circuit in prostate cancer mediates castration resistance. Cancer Cell.

[20] Beltran H (2019). The role of lineage plasticity in prostate cancer therapy resistance. Clin. Cancer Res..

[21] Liu, Y. et al. The androgen receptor regulates a druggable translational regulon in advanced prostate cancer. *Sci. Transl. Med*. **11**, eaaw4993 (2019). 10.1126/scitranslmed.aaw4993 PMC6746573 31366581

[22] Hu R (2012). Distinct transcriptional programs mediated by the ligand-dependent full-length androgen receptor and its splice variants in castration-resistant prostate cancer. Cancer Res..

[23] Frigo DE (2009). Induction of Kruppel-like factor 5 expression by androgens results in increased CXCR4-dependent migration of prostate cancer cells in vitro. Mol. Endocrinol..

[24] Lee MY (2009). KLF5 enhances SREBP-1 action in androgen-dependent induction of fatty acid synthase in prostate cancer cells. Biochem J..

[25] Li, J. et al. KLF5 is crucial for androgen-AR signaling to transactivate genes and promote cell proliferation in prostate cancer cells. *Cancers (Basel)* **12**, 748 (2020). 10.3390/cancers12030748 PMC7140031 32245249

[26] Thalmann GN (1994). Androgen-independent cancer progression and bone metastasis in the LNCaP model of human prostate cancer. Cancer Res.

[27] Li Y (2013). Androgen receptor splice variants mediate enzalutamide resistance in castration-resistant prostate cancer cell lines. Cancer Res.

[28] Zhang X (2016). Identification of focally amplified lineage-specific super-enhancers in human epithelial cancers. Nat. Genet.

[29] Zhang B (2021). Acetylation of KLF5 maintains EMT and tumorigenicity to cause chemoresistant bone metastasis in prostate cancer. Nat. Commun..

[30] Kumar A (2016). Substantial interindividual and limited intraindividual genomic diversity among tumors from men with metastatic prostate cancer. Nat. Med.

[31] Robinson D (2015). Integrative clinical genomics of advanced prostate cancer. Cell.

[32] Liberzon A (2015). The molecular signatures database (MSigDB) hallmark gene set collection. Cell Syst..

[33] Subramanian A (2005). Gene set enrichment analysis: a knowledge-based approach for interpreting genome-wide expression profiles. Proc. Natl Acad. Sci. USA.

[34] Creighton CJ (2006). Activation of mitogen-activated protein kinase in estrogen receptor alpha-positive breast cancer cells in vitro induces an in vivo molecular phenotype of estrogen receptor alpha-negative human breast tumors. Cancer Res.

[35] Henry GH (2018). A cellular anatomy of the normal adult human prostate and prostatic urethra. Cell Rep..

[36] Davies A, Zoubeidi A, Selth LA (2020). The epigenetic and transcriptional landscape of neuroendocrine prostate cancer. Endocr. Relat. Cancer.

[37] Abida W (2019). Genomic correlates of clinical outcome in advanced prostate cancer. Proc. Natl Acad. Sci. USA.

[38] Beltran, H. et al. Divergent clonal evolution of castration-resistant neuroendocrine prostate cancer. *Nat Med.* **22,** 298–305 (2016). 10.1038/nm.4045 PMC4777652 26855148

[39] Akamatsu S (2015). The placental gene PEG10 promotes progression of neuroendocrine prostate cancer. Cell Rep..

[40] Grosset AA, Poirier F, Gaboury L, St-Pierre Y (2016). Galectin-7 expression potentiates HER-2-positive phenotype in breast cancer. PLoS ONE.

[41] Hong J (2012). Regulation of ERBB2 receptor by t-DARPP mediates trastuzumab resistance in human esophageal adenocarcinoma. Cancer Res.

[42] Ji H (2016). EGFR phosphorylates FAM129B to promote Ras activation. Proc. Natl Acad. Sci. USA.

[43] Nyquist MD (2013). TALEN-engineered AR gene rearrangements reveal endocrine uncoupling of androgen receptor in prostate cancer. Proc. Natl Acad. Sci. USA.

[44] Puca L (2018). Patient derived organoids to model rare prostate cancer phenotypes. Nat. Commun..

[45] Geeleher P, Cox NJ, Huang RS (2014). Clinical drug response can be predicted using baseline gene expression levels and in vitro drug sensitivity in cell lines. Genome Biol..

[46] Network CGAR (2015). The molecular taxonomy of primary prostate cancer. Cell.

[47] Mu P (2017). SOX2 promotes lineage plasticity and antiandrogen resistance in TP53- and RB1-deficient prostate cancer. Science.

[48] Mellinghoff IK (2004). HER2/neu kinase-dependent modulation of androgen receptor function through effects on DNA binding and stability. Cancer Cell.

[49] Gao S (2016). ErbB2 signaling increases androgen receptor expression in abiraterone-resistant prostate cancer. Clin. Cancer Res.

[50] Cai C (2011). Androgen receptor gene expression in prostate cancer is directly suppressed by the androgen receptor through recruitment of lysine-specific demethylase 1. Cancer Cell.

[51] Whang YE (2013). A phase II study of lapatinib, a dual EGFR and HER-2 tyrosine kinase inhibitor, in patients with castration-resistant prostate cancer. Urol. Oncol..

[52] Shiota M (2015). Inhibition of the HER2-YB1-AR axis with lapatinib synergistically enhances enzalutamide anti-tumor efficacy in castration resistant prostate cancer. Oncotarget.

[53] Paltoglou, S. et al. Novel androgen receptor co-regulator GRHL2 exerts both oncogenic and anti-metastatic functions in prostate cancer. *Cancer Res.* **77**, 417–3430 (2017). 10.1158/0008-5472.CAN-16-1616 PMC5497757 28473532

[54] Chan SC (2015). Targeting chromatin binding regulation of constitutively active AR variants to overcome prostate cancer resistance to endocrine-based therapies. Nucleic Acids Res.

[55] Zheng R (2019). Cistrome Data Browser: expanded datasets and new tools for gene regulatory analysis. Nucleic Acids Res.

[56] Singh AA (2019). Optimized ChIP-seq method facilitates transcription factor profiling in human tumors. Life Sci. Alliance.

[57] Li H, Durbin R (2009). Fast and accurate short read alignment with Burrows–Wheeler transform. Bioinformatics.

[58] Quinlan AR, Hall IM (2010). BEDTools: a flexible suite of utilities for comparing genomic features. Bioinformatics.

[59] Zhang Y (2008). Model-based analysis of ChIP-Seq (MACS). Genome Biol..

[60] Glumac PM (2018). The identification of a novel antibody for CD133 using human antibody phage display. Prostate.

[61] Simon, R., Mirlacher, M. & Sauter, G. in *Tissue Microarrays: Methods and Protocols* (ed Ronald Simon) 113–126 (Humana Press, 2010). 10.1007/978-1-60761-806-5_12 20690058

[62] Chaturvedi A, Hoffman LM, Welm AL, Lessnick SL, Beckerle MC (2012). The EWS/FLI oncogene drives changes in cellular morphology, adhesion, and migration in Ewing sarcoma. Genes Cancer.

[63] Kim D, Langmead B, Salzberg SL (2015). HISAT: a fast spliced aligner with low memory requirements. Nat. Methods.

[64] Liao Y, Smyth GK, Shi W (2013). The Subread aligner: fast, accurate and scalable read mapping by seed-and-vote. Nucleic Acids Res.

[65] Cunningham F (2019). Ensembl 2019. Nucleic Acids Res.

[66] McCarthy DJ, Chen Y, Smyth GK (2012). Differential expression analysis of multifactor RNA-seq experiments with respect to biological variation. Nucleic Acids Res.

[67] Robinson MD, McCarthy DJ, Smyth G (2010). K. edgeR: a Bioconductor package for differential expression analysis of digital gene expression data. Bioinformatics.

[68] Li Y (2011). Intragenic rearrangement and altered RNA splicing of the androgen receptor in a cell-based model of prostate cancer progression. Cancer Res.

[69] Gao D (2014). Organoid cultures derived from patients with advanced prostate cancer. Cell.

