# Supplementary material for: Opposing transcriptional programs of KLF5 and AR emerge during therapy for advanced prostate cancer
Source: Nat Commun. 2021 Nov 4;12:6377. doi: 10.1038/s41467-021-26612-1 (PMC8568894; doi:10.1038/s41467-021-26612-1)
Supplement: Supplementary file 1 — Supplementary Information [file 41467_2021_26612_MOESM1_ESM.pdf]

Supplementary Methods
Supplementary Table 1
Supplemental Figures 1-21
Supplementary References

Opposing transcriptional programs of KLF5 and AR emerge during therapy for advanced prostate cancer

Meixia Che, Aashi Chaturvedi, Sarah Munro, *et al.*

Supplementary Methods

Cell Line Authentication and Mycoplasma Testing Procedures

ATCC ensures authenticity of LNCaP, VCaP, 22RV1, DU145, PC3 and NCI-H660 cell lines using short tandem repeat analysis. Authentication of the R1-AD1 cell line was performed by sequence-based validation of an AR H874Y point mutation and authentication of the R1-D567 cell line was performed by PCR-based validation of a targeted genomic deletion of AR exons 5-7. Authentication of LNCaP-derived sub-lines was performed by sequence-based validation of an AR T878A point mutation. Aliquots of cell culture supernatants from cells in active culture were evaluated regularly for mycoplasma contamination using a PCR-based method as described [1]. All cell line experiments were performed within 2-3 months of resuscitation of frozen cell stocks prepared within 3 passages of receipt.

Plasmids

A lentivirus expression vector encoding AR (or GFP as control) under control of a SV40 promoter has been described [2].

Co-Immunoprecipitation

R1-AD1 or R1-D567 cells were transfected with a plasmid encoding FLAG tagged KLF5 (Addgene, Plasmid #40918) using electroporation as described [2]. Cells were washed twice with 1X PBS and lysed in 1 mL IP Lysis Buffer (50 mM Tris-HCl, pH 7.5, 150 mM NaCl, 1% (v/v) NP-40, 0.5% (v/v) sodium deoxycholate supplemented with Roche Complete protease inhibitor) post 48 h transfection. The cell lysate was transferred to a pre-chilled Dounce homogenizer on ice with a type B pestle and was homogenized for 10 strokes. Debris was pelleted via centrifugation (12,000x g, 10 min) at 4 °C and the supernatant was transferred to a fresh tube. Lysates of 500 μg of total protein in 1 mL was used for each reaction. Anti-FLAG antibody (5 μg, Sigma) or a control anti-mouse IgG (5 μg, Santa Cruz) was added to pre-cleared lysate and was mixed on rotator for 1 h at 4 °C. A 50 μL slurry of Protein G-agarose was added to each reaction and rotated overnight at 4 °C. The agarose beads were subjected to centrifuge and washed 3 times with 1 mL IP Lysis Buffer for 5 min. The immunoprecipitated protein from each sample was eluted with 50 μL of 1 x Laemmli Buffer. A 10 μL sample of the eluate was used for Western blot.

Gene Set Enrichment Analysis (GSEA) of Public Gene Expression Databases

The Co-expression tool in cBioPortal [3,4] was used to download lists of genes and their correlations with KLF5 expression in RNA-seq data from 150 CRPC metastatic biopsies (the SU2C study [5]) or RNA-seq data from 176 tumors from autopsy of 63 patients that died of metastatic prostate cancer (the FHCRC study [6]) on 12/18/2017. Co-expression lists were ordered by Pearson score and trimmed to the top 1000 or bottom 1000 correlated genes. Intersecting the top and bottom genes in the lists derived from the SU2C or FHCRC studies

provided a set of 100 genes positively-correlated with KLF5 expression and a set of 109 genes negatively-correlated with KLF5 expression in both clinical CRPC datasets. GSEA was performed using GSEA v3.0 (Broad Institute, [7]). Genes were ranked using the Signal2Noise metric and GSEA was performed against 1000 random gene set permutations.

Correlation of KLF5 with AR and NEPC Scores

The Plots tool in cBioPortal [3,4] was used to analyze the relationships between KLF5 mRNA expression (mRNA expression, FPKM capture) and Clinical Attributes of AR Score (Capture) or NEPC Score (Capture) in the Metastatic Prostate Adenocarcinoma (SU2C/PCF Dream Team) dataset [8] on 10/9/2019.

Gene Sets for Determining Activity Scores

All gene sets used for determining activity scores are listed in Supplementary Data 1. The cell cycle progression gene list and AR activity gene list have been described [6]. The 37 KLF5 target genes were selected by the intersect of the three GSEA leading edges where the CRPC_KLF5_pos gene signature was tested for enrichment in RNA-seq data from R1-D567 cells and R1-AD1 cells cultured under DHT conditions and vehicle (ethanol) conditions. These GSEA analyses are illustrated in Supplementary Figs. 6b & 15d.

Gene Ontology Analysis

We tested for enrichment of differentially expressed genes with positive fold change (log2FC > 0) for each comparison against the Gene Ontology (GO) database [9,10] using the goana and topGo functions in the edgeR package. This analysis was performed for two sets of data: the full positive differential expression gene lists for each differential expression comparison and the subset of the genes in the differential expression lists annotated with KLF5/ARCommon ChIP-seq peak associations (within +/- 50 kb).

Prediction of Patient Response to Lapatinib

RNA-seq data from 90 samples analyzed in the Prostate Cancer Medically Optimized Genome-Enhanced Therapy (PROMOTE) study [11] were obtained from dbGap (phs001141.v1.p1). Sequence quality control metrics were calculated using FASTQC (v0.11.8) (http://www.bioinformatics.babraham.ac.uk/projects/fastqc). The illumina sequencing adaptors were removed using Trim Galore (v0.6.0) (https://www.bioinformatics.babraham.ac.uk/projects/trim_galore/). Next, raw RNA-Seq reads were mapped to the human reference genome (hg19) using TopHat (v2.0.6) 1 with bowtie1 aligner options. Raw reads count for genes were calculated using featureCounts 2 package (v1.5.2) with parameter “-p” for paired-end reads and GENCODE human gene model (version 19) as the reference gene model. The gene expression values were measured by the normalized value of fragments per kilobase of exon model per million fragments mapped (FPKM) 3 using in-house Python script.

RNA-seq data from 212 samples analyzed by exome capture in the Prostate Cancer Foundation Stand-Up-To-Cancer East Coast Study (SU2C-EC) [5,8] were downloaded from cBioPortal [3,4] on 1/4/2020 using the following link: http://download.cbioportal.org/prad_su2c_2019.tar.gz. RNA-seq data from 551 samples analyzed in The Cancer Genome Atlas Prostate Adenocarcinoma Study (TCGA-PRAD) [12] was downloaded from UCSC Xena [13] on 6/21/2019 using the following link: https://gdc.xenahubs.net/download/TCGA-PRAD/Xena_Matrices/TCGA-PRAD.htseq_fpkm.tsv.gz. Cancer cell line drug sensitivity data was obtained from the Cancer Therapeutics Response Portal Version 2 (CTRPv2) cancer cell line screen [14-16], downloaded on 11/7/2017 using the following link: ftp://caftpd.nci.nih.gov/pub/OCG-DCC/CTD2/Broad/CTRPv2.0_2015_ctd2_ExpandedDataset/CTRPv2.0_2015_ctd2_ExpandedDataset.zip and cancer cell line gene expression data was downloaded from the Cancer Cell Line Encyclopedia (CCLE) [17] on 11/7/2017 using the following link: https://data.broadinstitute.org/ccle/CCLE_RNAseq_081117.rpkm.gct. These datasets were used to predict lapatinib sensitivities for the patients in each study as described [18]. Briefly, a ridge regression was used to model measured lapatinib sensitivity in CTRPv2 cell lines using the gene expression data for those cell lines from CCLE. This regression model was then applied to RNA-seq gene expression data from PROMOTE, SU2C-EC, and TCGA-PRAD to predict lapatinib sensitivity in those patients. Pearson correlation coefficients were then calculated between predicted lapatinib sensitivities and KLF5 gene expression for each dataset. We also calculated Pearson correlation coefficients between predicted lapatinib sensitivities and a KLF5 activity score for each dataset. Analyses were performed using R version 3.6.1, RStudio version 1.2.5019, and the following R packages: readxl version 1.3.1, progress version 1.2.2, glmnet version 3.0.1 [19], sva version 3.34.0, doParallel version 1.0.15, biomaRt version 2.32.1 [20,21].

Supplementary Table 1: PCR Primer Sequences

Primer Name	Primer Sequence
KLK3 RT-PCR Forward	5'-AGGCCTTCCCTGTACACCAA
KLK3 RT-PCR Reverse	5'-GTCTTGGCCTGGTCATTTCC
KLF5 RT-PCR Forward (human)	5'-ACACCAGACCGCAGCTCCA
KLF5 RT-PCR Reverse (human)	5'-TCCATTGCTGCTGTCTGATTTGTAG
Klf5 RT-PCR Forward (mouse)	5'-GAGGACTCATACGGGCGAGA
Klf5 RT-PCR Reverse (mouse)	5'-CAGTTCTGGTGGCGCTTCAT
PPP1R1B RT-PCR Forward	5'-CAGCACTAAGTGAGCCTGGG
PPP1R1B RT-PCR Reverse	5'-CTTCCTCCTGGGAGATGCAG
ERBB2 RT-PCR Forward	5'-ACCAAGCTCTGCTCCACACT
ERBB2 RT-PCR Reverse	5'-ACTGGCTGCAGTTGACACAC
FAM129B RT-PCR Forward	5'-GCCAGCACATCGCAGAAAAA
FAM129B RT-PCR Reverse	5'-CGCATGCTGTTGAAGAGAGC
LGALS7 RT-PCR Forward	5'-CCTTCGAGGTGCTCATCATC
LGALS7 RT-PCR Reverse	5'-GAAGATCCTCACGGAGTCCA
ACTB RT-PCR Forward	5'-CTCTTCCAGCCTTCCTTCCT
ACTB RT-PCR Reverse	5'-AGCACTGTGTTGGCGTACAG
Gapdh RT-PCR Forward (mouse)	5'- CAATGAATACGGCTACAGCAAC
Gapdh RT-PCR Reverse (mouse)	5'- AGGGAGATGCTCAGTGTTGG

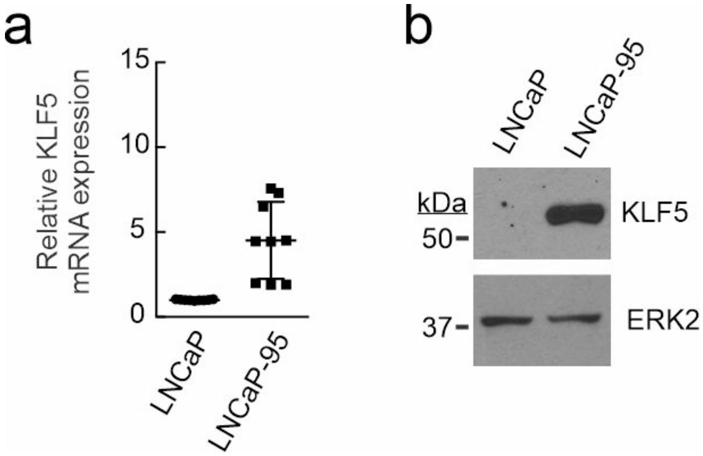

Supplementary Figure 1. KLF5 expression in LNCaP-95 cells. a KLF5 mRNA measured using quantitative RT-PCR in LNCaP and LNCaP-95 cells Data represent 3 independent biological replicates performed in technical triplicate (n=9). Individual data points are shown along with mean +/- 95% CI. **b** KLF5 protein measured by western blot in LNCaP and LNCaP-95 cells. ERK2 is a loading control.

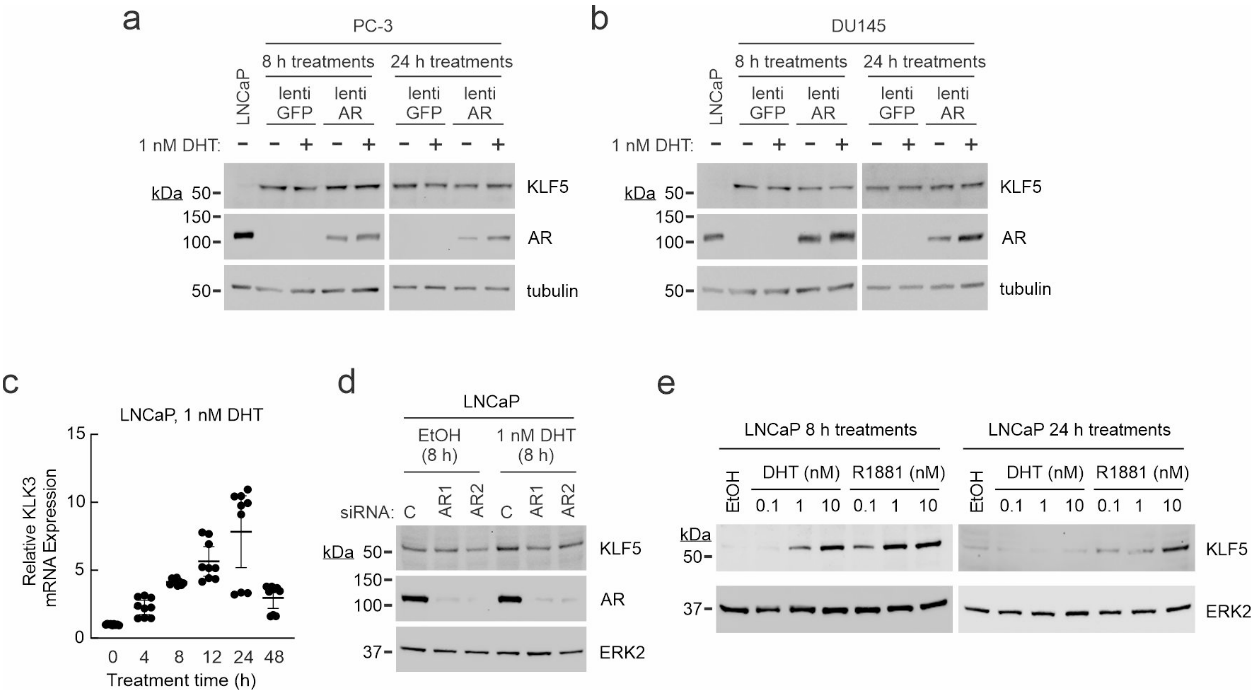

Supplementary Figure 2. Androgen regulation of KLF5. a KLF5 and AR protein measured by western blot in PC-3 cells infected with lentivirus encoding AR (or GFP as control), treated 8 or 24 hours with 1 nM DHT. One additional replicate experiment was performed that yielded a comparable result. **b** KLF5 and AR protein measured in DU145 cells infected and treated as in **a**. One additional replicate experiment was performed that yielded a comparable result. **c** KLK3 (encoding PSA) mRNA measured by RT-PCR in LNCaP cells treated with 1 nM DHT. n = 9, mean +/- 95% CI from 3 biological replicates in technical triplicate. **d** KLF5 and AR protein measured by western blot in LNCaP cells transfected with AR-targeted siRNA (AR1 and AR2) or control siRNA. One additional replicate experiment was performed that yielded a comparable result. **e** KLF5 protein measured by western blot in LNCaP cells treated with 0.1-10 nM DHT or R1881 (or ethanol as vehicle control) for 8 h or 24 h. One additional replicate experiment was performed that yielded a comparable result.

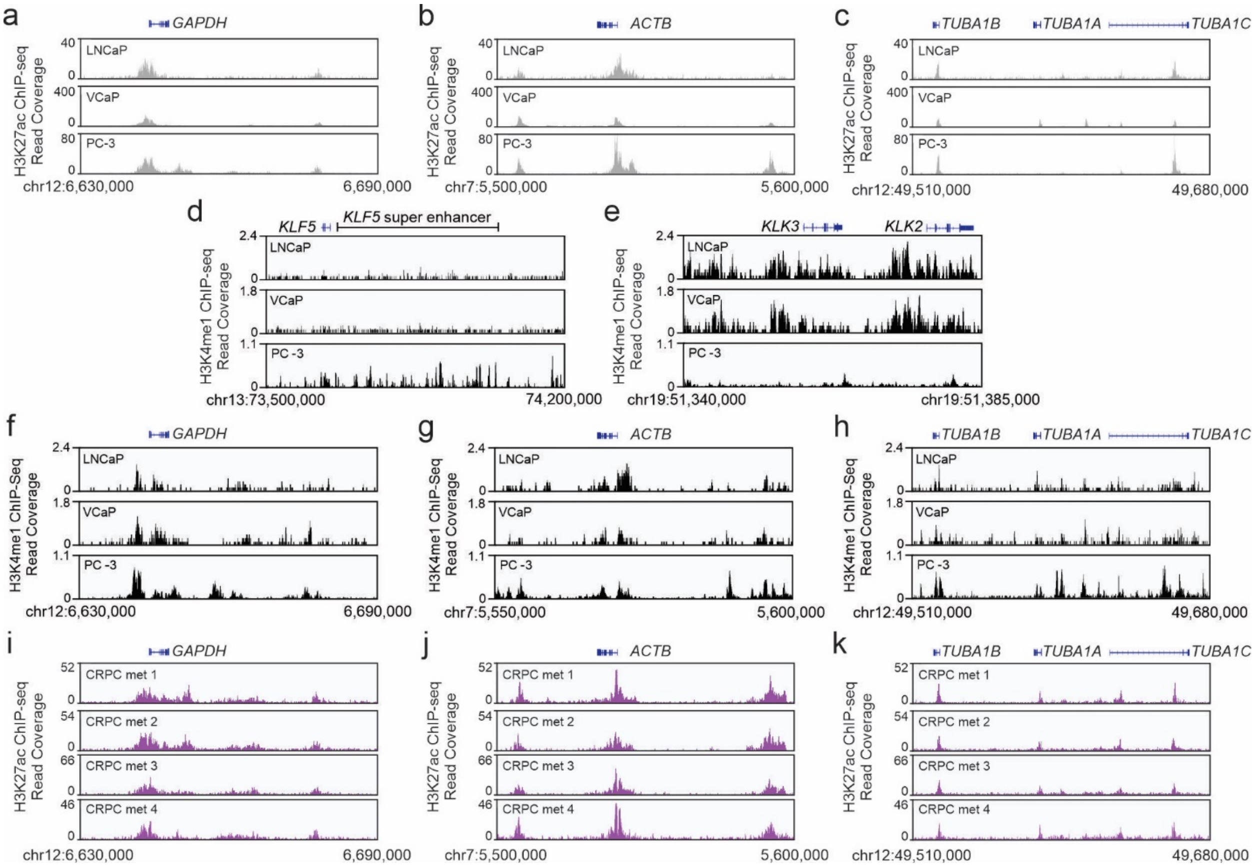

Supplementary Figure 3. H3K27ac and H3K4me1 ChIP-seq data at *KLF5*, *KLK2/3*, and housekeeping gene loci. a-c H3K27ac ChIP-seq data from LNCaP, VCaP, and PC-3 cells at genomic loci for housekeeping controls *GAPDH* (encodes glyceraldehyde 3-phosphate dehydrogenase), *ACTB* (encodes beta-actin), and *TUBA1A-C* (encode tubulin alpha-1A, -1B, and -1C chains). **d-h** H3K4me1 ChIP-seq data from LNCaP, VCaP, and PC-3 cells at genomic loci for *KLF5*, *KLK2/3*, *GAPDH*, *ACTB*, and *TUBA1A-C*. **i-k** H3K27ac ChIP-seq data from 4 clinical CRPC specimens at genomic loci for *GAPDH*, *ACTB*, and *TUBA1A-C*.

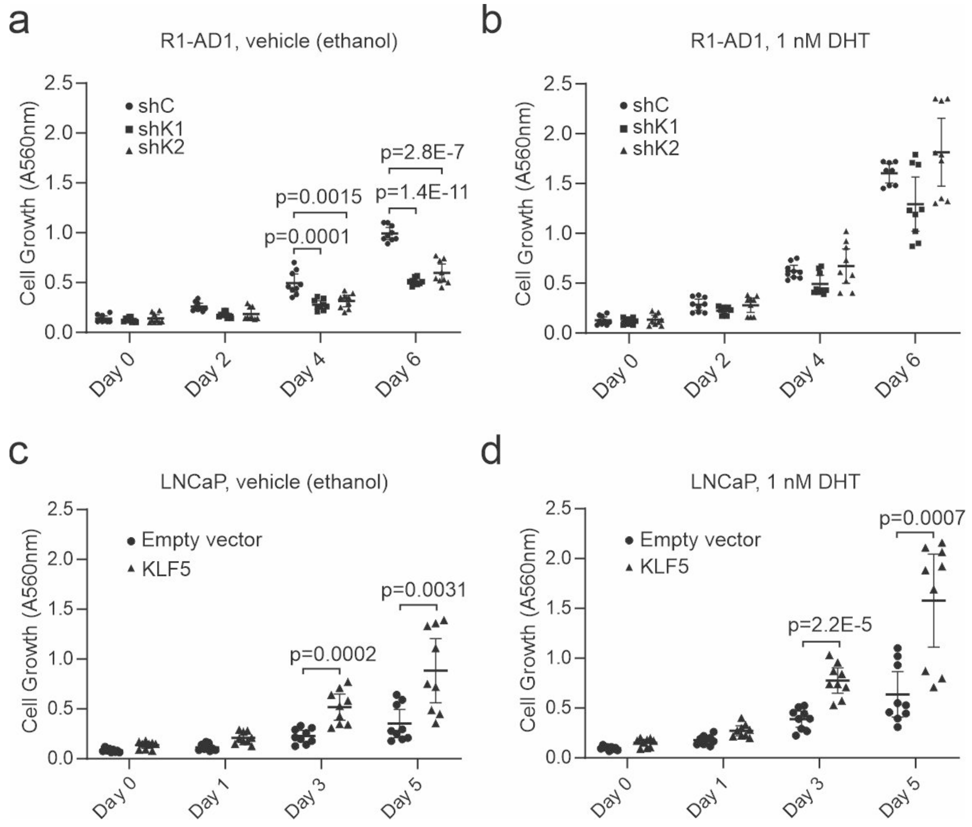

Supplementary Figure 4. Effects of KLF5 expression on cell growth in 2-dimensional (2D) growth assays. **a** R1-AD1 cells infected with lentivirus encoding shRNAs targeting KLF5 or control shRNA were seeded on tissue culture plates in medium supplemented with 10% charcoal-stripped (steroid depleted) medium containing 0.1% v/v ethanol. Cells were subjected to 2D growth assays by fixing and crystal violet staining at indicated time points. **b** 2D growth assays as in **a** with medium containing 1 nM dihydrotestosterone (DHT). **c** 2D growth assays as in **a** with LNCaP cells infected with empty lentivirus or lentivirus encoding KLF5. **d** 2D growth assays as in **c** with medium containing 1 nM DHT. Data represent 9 independent biological replicates (n=9). Individual data points are shown along with mean +/- 95% CI. P-values were determined using unpaired 2-sided t-tests.

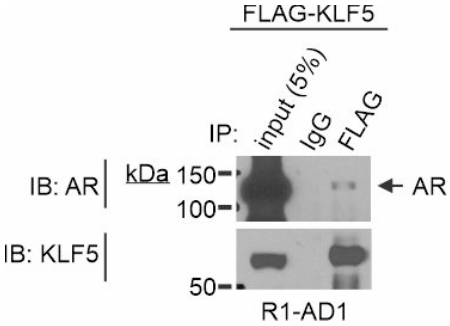

Supplementary Figure 5. Co-Immunoprecipitation of AR and FLAG-KLF5. R1-AD1 cells expressing FLAG-KLF5 were lysed and subjected to immunoprecipitation with anti-FLAG antibodies or IgG control. Immunoprecipitates were analyzed by western blot using antibodies specific for AR and KLF5. The input lane represents 5% of the lysate. Two additional replicate experiments were performed that yielded comparable results.

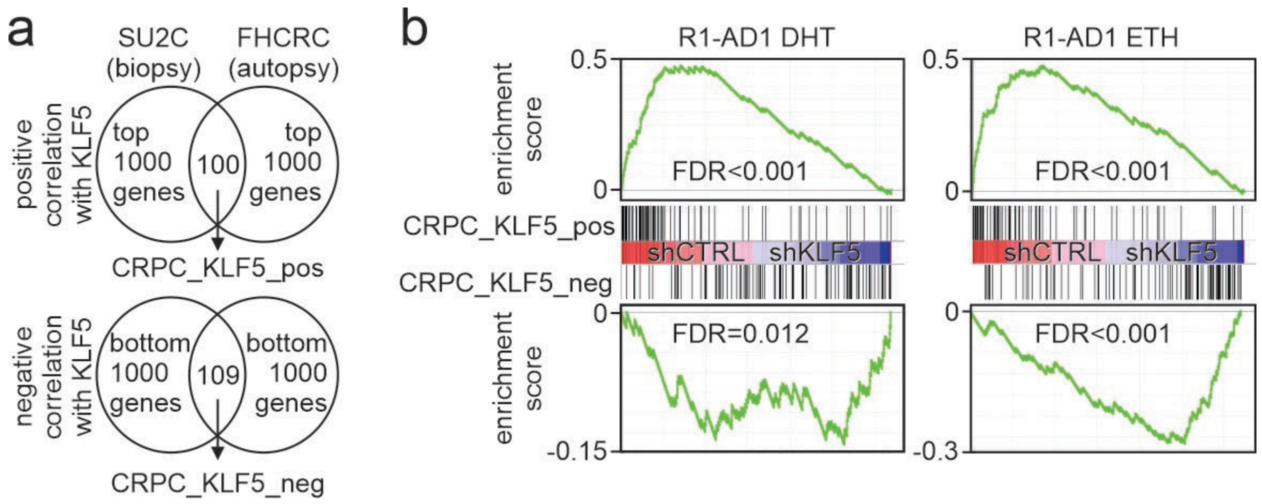

Supplementary Figure 6. Gene set enrichment analysis of KLF5-correlated gene sets from clinical CRPC specimens in RNA-seq data from R1-AD1 KLF5 knock-down cells.
a The 100-gene CRPC_KLF5_pos gene set was derived from the intersection of the top 1000 genes positively correlated with KLF5 expression in the AACR-PCF Stand-Up-To-Cancer study of CRPC biopsies (SU2C) or Fred Hutchinson Cancer Research Center study of CRPC autopsies (FHCRC). The 109-gene CRPC_KLF5_neg gene set was derived using the same intersection strategy, but with the bottom 1000 genes that correlated with KLF5 expression.
b Gene set enrichment analysis was used to test enrichment of the CRPC_KLF5_pos and CRPC_KLF5_neg gene sets in RNA-seq data from R1-AD1 cells cultured in the presence of dihydrotestosterone (DHT) or vehicle control (ethanol, ETH), ordered by differential expression based on infection with lentivirus encoding KLF5 (shKLF5) or control shRNA (shCTRL).

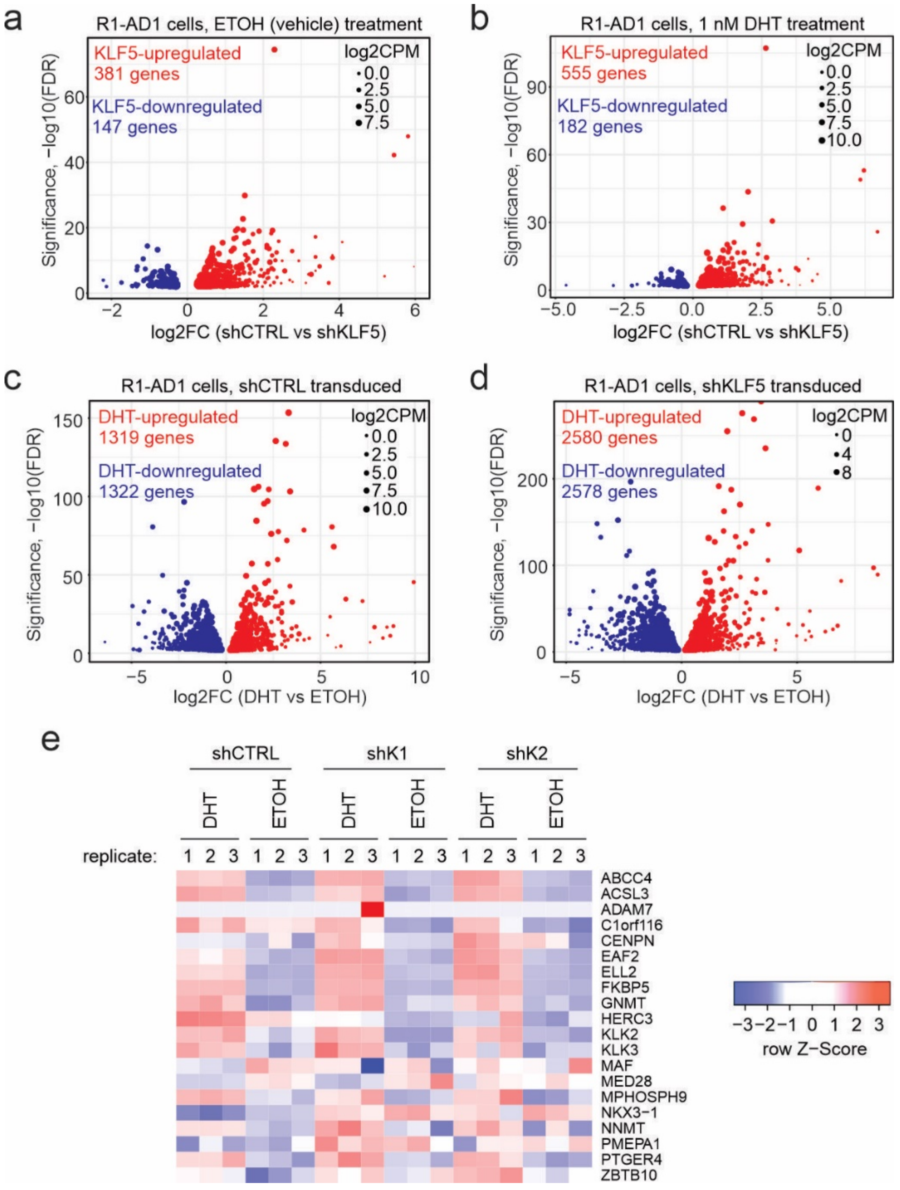

Supplementary Figure 7. KLF5- and androgen-regulated genes in R1-AD1 cells.
a Differentially-expressed genes in R1-AD1 cells infected with lentivirus encoding KLF5 shRNA (shKLF5) vs. control shRNA (shCTRL). Data reflect RNA-seq data collected from 3 independent biological replicate experiments with cells that were infected with shCTRL or 2 independent shKLF5 constructs. Cells in **a** were cultured in medium supplemented with 10% charcoal-stripped (steroid-depleted) serum (10% CSS) plus vehicle control (ethanol, ETOH). **b** Differentially expressed genes in R1-AD1 cells infected and analyzed exactly as in **a** with cells cultured in 10% CSS medium supplemented with 1 nM dihydrotestosterone (DHT). **c** Differentially-expressed genes in shCTRL-infected R1-AD1 cells cultured in medium containing 10% CSS medium supplemented with ETOH vs. 1 nM DHT. **d** Differentially-expressed genes in shKLF5-infected R1-AD1 cells cultured +/- DHT as in **c**. Sizes of dots in plots reflect the log2 of counts per million (CPM) measured for that gene. **e** Heatmap of RNA-seq gene expression data for a 20-gene panel of clinically-relevant AR target genes.

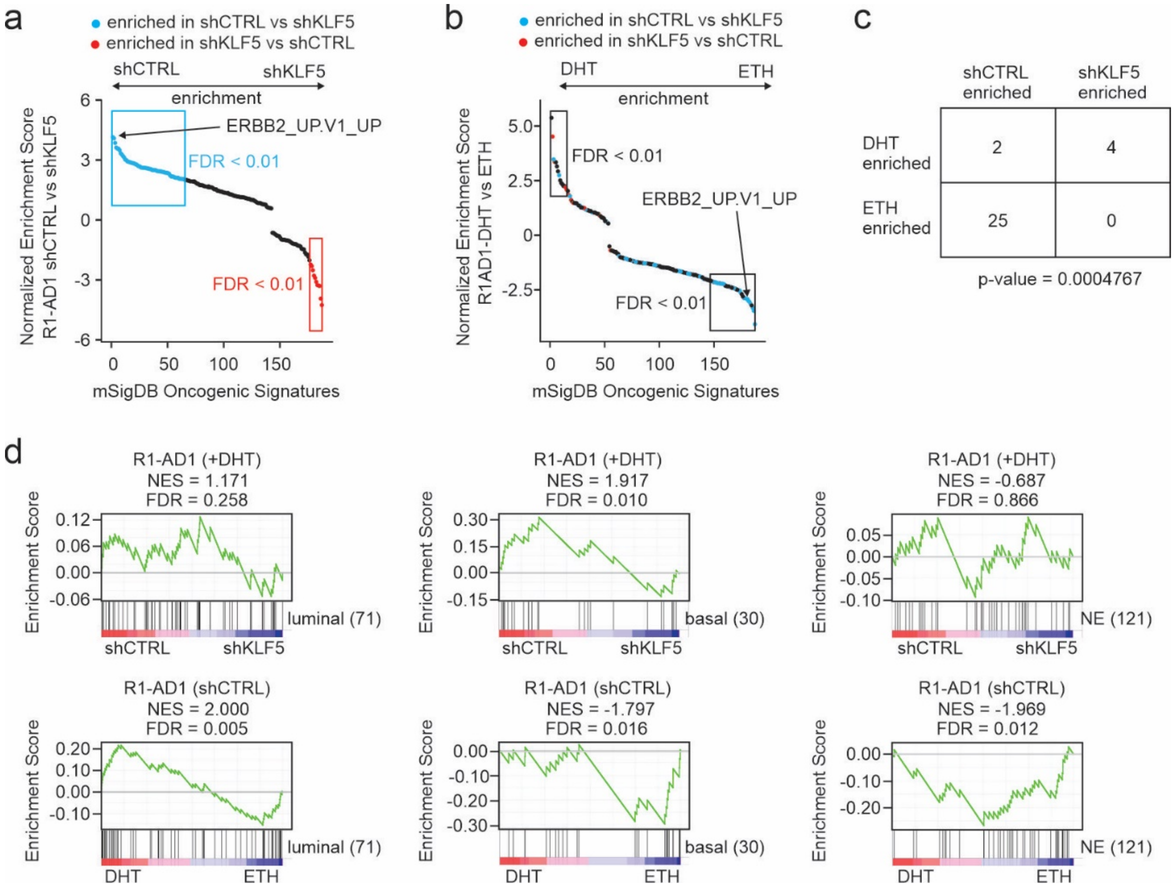

Supplementary Figure 8. GSEA Oncogenic Signatures and Fisher's exact test contingency table. **a** Normalized enrichment scores for all 189 MSigDB Oncogenic Signatures derived from gene set enrichment analysis (GSEA) using R1-AD1 gene expression data reflecting KLF5 activity (differential expression under shCTRL vs. shKLF5). **b** GSEA-derived normalized enrichment scores as in **a** using R1-AD1 gene expression data reflecting AR activity (differential expression in ETH vs. DHT). Oncogenic Signatures are colored blue or red based on whether they were positively- or negatively-enriched in **a** with FDR<0.01. **c** Contingency table indicating the number of mSigDB oncogenic signatures that were DHT enriched and shCTRL enriched, DHT enriched and shKLF5 enriched, ETH enriched and shCTRL enriched, or ETH enriched and shKLF5 enriched. P-value is from 2-sided Fisher's exact test. **d** GSEA testing enrichment of luminal, basal, and neuroendocrine (NE) gene signatures in R1-AD1 data reflecting active KLF5 (shCTRL vs. shKLF5) or active AR (DHT vs ETH) as indicated. FDR-corrected P-values were provided in GSEA output.

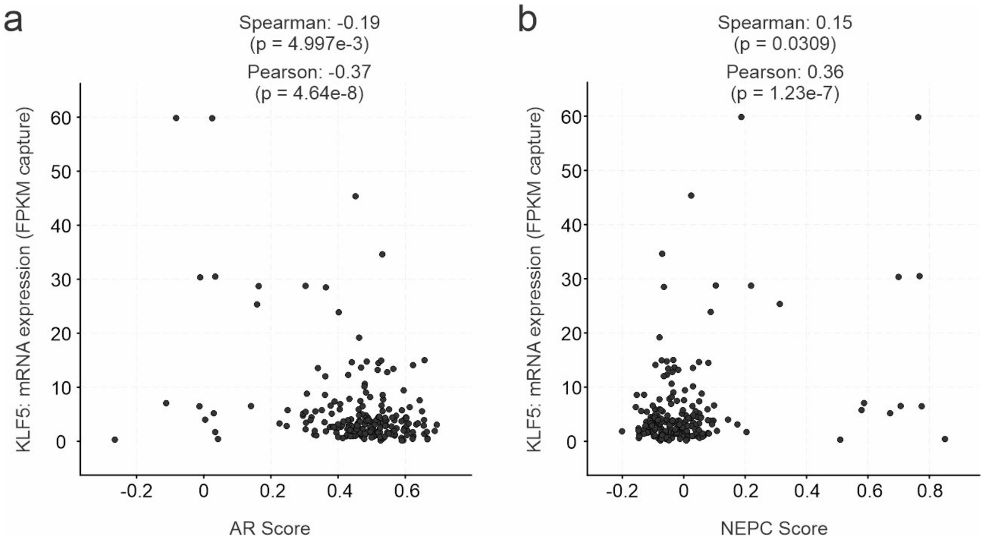

Supplementary Figure 9. Correlation of KLF5 expression with AR and NEPC activity scores. **a** Plot of KLF5 mRNA expression in fragments per kb per million mapped reads (FPKM) vs. an AR activity score. Plots were developed using data from a set of 209 CRPC tissues subjected to RNA-seq using hybrid capture. P-values are from 2-sided Spearman rank correlation and Pearson correlation tests as indicated. **b** Plot of KLF5 mRNA expression (in FPKM) vs. a neuroendocrine prostate cancer (NEPC) score developed from the same samples as in **a**. Plots were generated in cBioPortal.

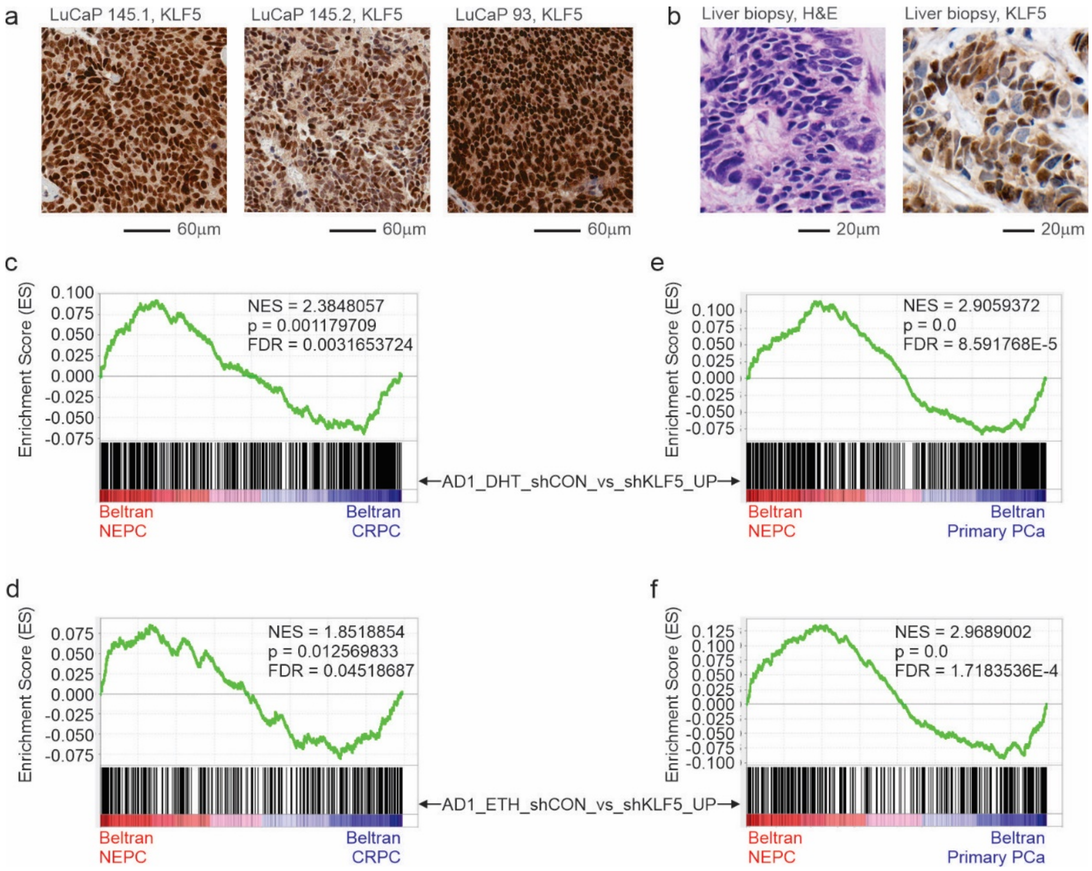

Supplementary Figure 10. KLF5 expression and target genes in clinical neuroendocrine CRPC (NEPC). a Immunohistochemistry (IHC) staining of NEPC patient derived xenograft (PDX) tissues with a KLF5 antibody. Images represent one TMA spot out of nine stained for KLF5 in each tissue, with similar results. **b** Hematoxylin and eosin (H&E, left) and IHC (right) staining of a NEPC metastatic biopsy (liver) with a KLF5 antibody. Images represent one KLF5 IHC stain performed on this tissue biopsy. **c** Enrichment of the AD1_DHT_shCON_vs_shKLF5_UP get set in gene expression data rank-ordered based on differential expression in clinical NEPC vs. CRPC tissues. Unadjusted and FDR-corrected P-values were provided in GSEA output. **d** Enrichment of the AD1_DHT_shCON_vs_shKLF5_UP get set in gene expression data rank-ordered based on differential expression in clinical NEPC vs. primary prostate cancer (PCa) tissues. Unadjusted and FDR-corrected P-values were provided in GSEA output. **e** Enrichment of the AD1_ETH_shCON_vs_shKLF5_UP get set in gene expression data rank-ordered based on differential expression in clinical NEPC vs. CRPC tissues. Unadjusted and FDR-corrected P-values were provided in GSEA output. **f** Enrichment of the AD1_ETH_shCON_vs_shKLF5_UP get set in gene expression data rank-ordered based on differential expression in clinical NEPC vs. primary prostate cancer (PCa) tissues. Unadjusted and FDR-corrected P-values were provided in GSEA output.

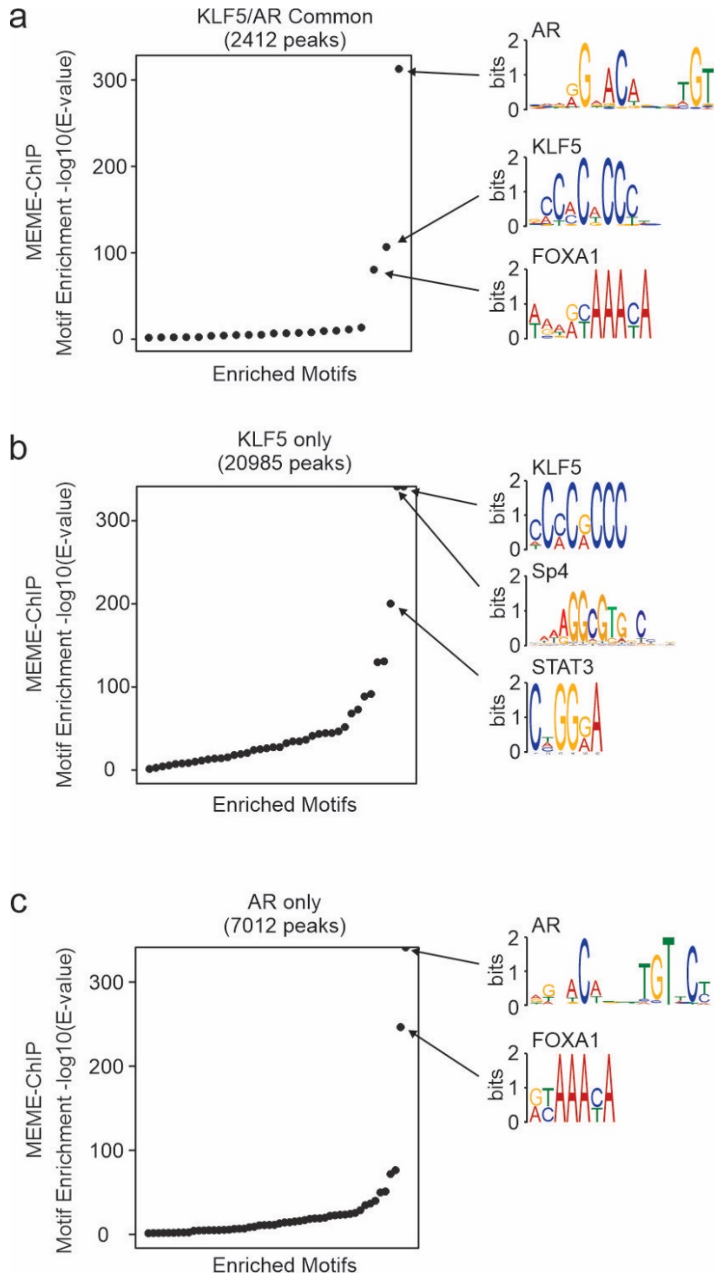

Supplementary Figure 11. Motif enrichment at AR and KLF5 peaks. a Motifs enriched at 2,412 KLF5/AR common peaks are plotted based on motif enrichment score determined by MEME-ChIP. Motifs with outlier enrichment score are indicated. **b** Motifs enriched at 20,985 KLF5 only peaks plotted as in **a**. **c** Motifs enriched at 7012 AR only peaks plotted as in **a**.

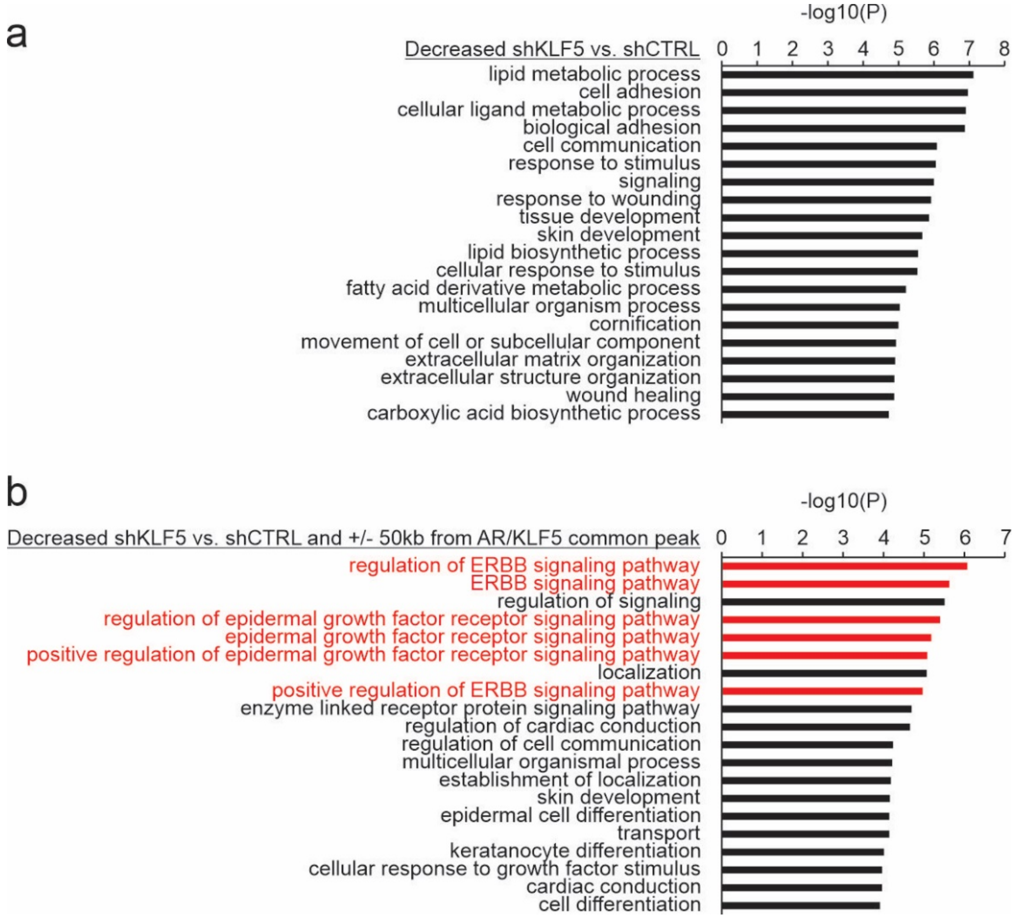

Supplementary Figure 12. Gene Ontology (GO) analysis of KLF5 target genes. a GO terms enriched for genes that display differential expression in RNA-seq data from R1-AD1 cells infected with lentivirus harboring shRNA targeted to KLF5 (shKLF5) vs. control shRNA (shCTRL). P-values are from output of the goana and topGo functions in edgeR. **b** GO terms enriched for genes located within +/- 50 kb from a shared KLF5/AR common peak and displaying differential expression in RNA-seq data from R1-AD1 cells infected with lentivirus harboring shRNA targeted to KLF5 (shKLF5) vs. control shRNA (shCTRL). GO terms associated with ERBB/epidermal growth factor receptor signaling pathways are highlighted in red. P-values are from output of the goana and topGo functions in edgeR.

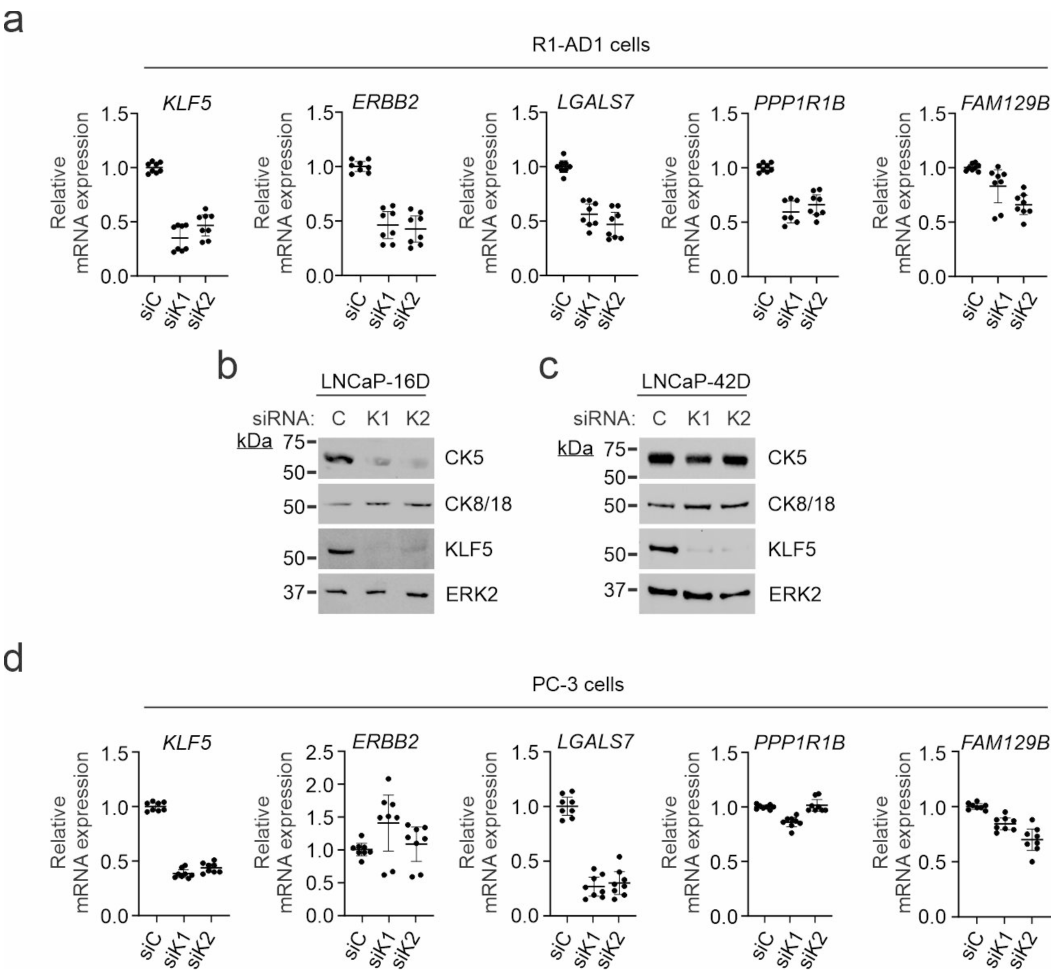

Supplementary Figure 13. Validation of KLF5 Target Genes. a mRNA levels of indicated targets were measured by RT-PCR in R1-AD1 cells transfected with KLF5-targeted siRNAs (siK1 and siK2) or control siRNA (siC) and maintained in androgen-depleted medium. Data are presented as mean +/- 95% CI with individual data points shown for 4 biological replicate experiments each performed in technical duplicate (n=8). **b,c** Cytokeratin-5 (CK5), Cytokeratin-8/18 (CK8/18), and KLF5 protein measured by western blot in LNCaP-16D and LNCaP-42D cells transfected and treated as in **a**. One additional replicate experiment was performed in each of LNCaP-16D and LNCaP-42D that yielded comparable results. **d** mRNA levels of indicated targets were measured by RT-PCR in PC-3 cells transfected and treated as in **a**. Data are presented as mean +/- 95% CI with individual data points shown for 4 biological replicate experiments each performed in technical duplicate (n=8)

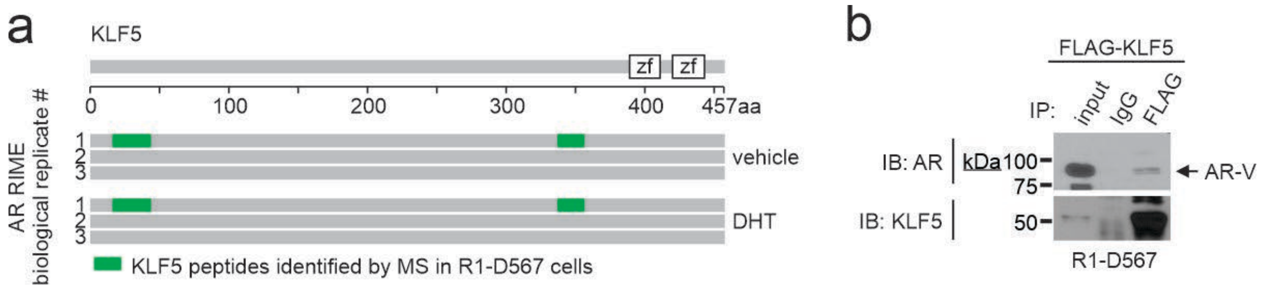

Supplementary Figure 14. KLF5 interaction with AR variant (AR-V) protein ARv567es. a KLF5 peptides identified in AR immunoprecipitates generated using rapid immunoprecipitation and mass spectrometry of endogenous proteins (RIME). **b** R1-D567 cells were transfected with a vector encoding FLAG-tagged KLF5. Proteins immunoprecipitated (IP) with FLAG antibody or control IgG were analyzed by immunoblot (IB) with antibodies specific for the AR N-terminal domain or KLF5. Pull-down of AR-V protein is indicated.

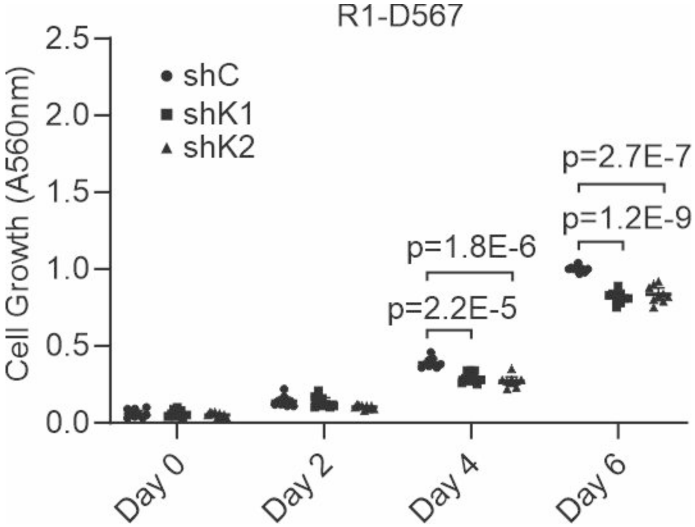

Supplementary Figure 15. Effects of KLF5 expression on R1-D567 cell growth in 2-dimensional (2D) growth assays. R1-D567 cells infected with lentivirus encoding shRNAs targeting KLF5 or control shRNA were seeded on tissue culture plates in medium supplemented with 10% charcoal-stripped (steroid depleted) medium. Cells were subjected to 2D growth assays by fixing and crystal violet staining at indicated time points. Data represent 9 independent biological replicates (n=9). Individual data points are shown along with mean +/- 95% CI. P-values were determined using unpaired 2-sided t-tests.

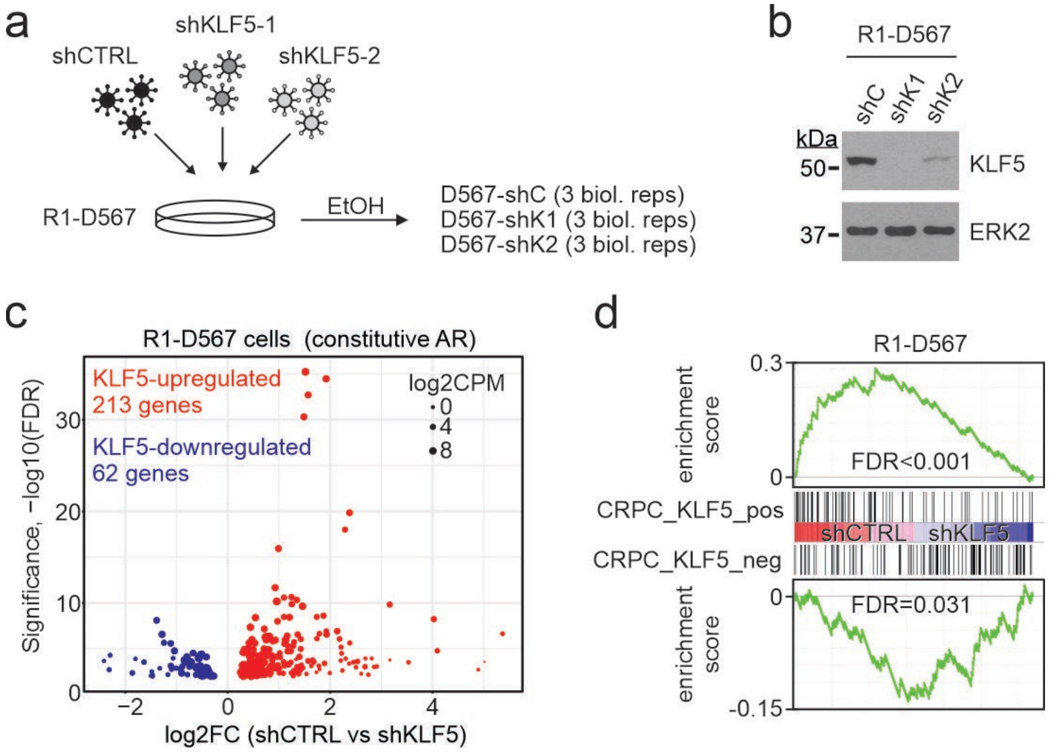

Supplementary Figure 16. Identification of KLF5 target genes in R1-D567 cells using RNA-seq. **a** Schematic of workflow for infection of R1-D567 cells with lentivirus encoding control shRNA (shCTRL) or two independent shRNAs targeting KLF5 (shKLF5). **b** Western blot of lysates from cells infected as in **a**. Blots were probed with antibodies specific for KLF5 or ERK2 as loading control. Two additional replicate experiments were performed that yielded comparable results. **c** Volcano plots of differentially-expressed genes in R1-D567 cells infected with lentivirus and analyzed by RNA-seq as in **a**. **d** Gene set enrichment analysis of KLF5-correlated clinical gene sets (CRPC_KLF5_pos and CRPC_KLF5_neg) in RNA-seq data from R1-D567 cells cultured as in **a**. FDR-corrected P-values were provided in GSEA output.

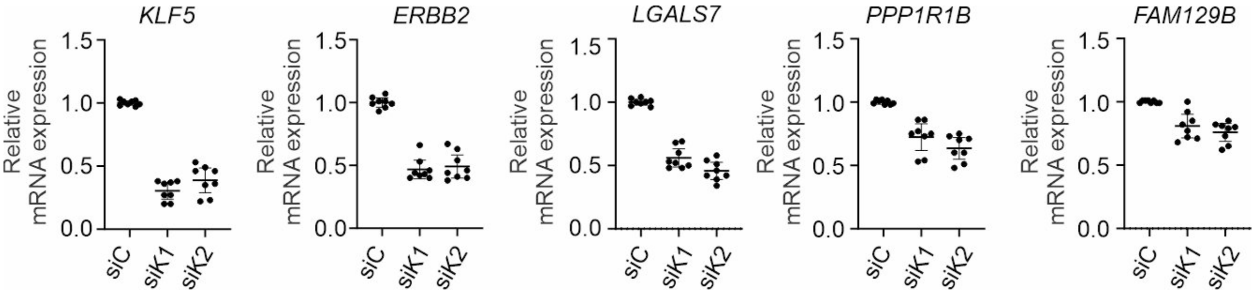

Supplementary Figure 17. Validation of KLF5 Target Genes in R1-D567 Cells. R1-D567 cells were transiently transfected with KLF5-targeted siRNAs (siK1 and siK2) or control siRNA (siC) and maintained in androgen-replete medium. mRNA expression levels of *KLF5*, *ERBB2*, *LGALS7*, *PPP1R1B*, and *FAM129B* were analyzed by RT-PCR. Data are presented as mean +/- 95% CI with individual data points shown for 4 biological replicate experiments each performed in technical duplicate (n=8).

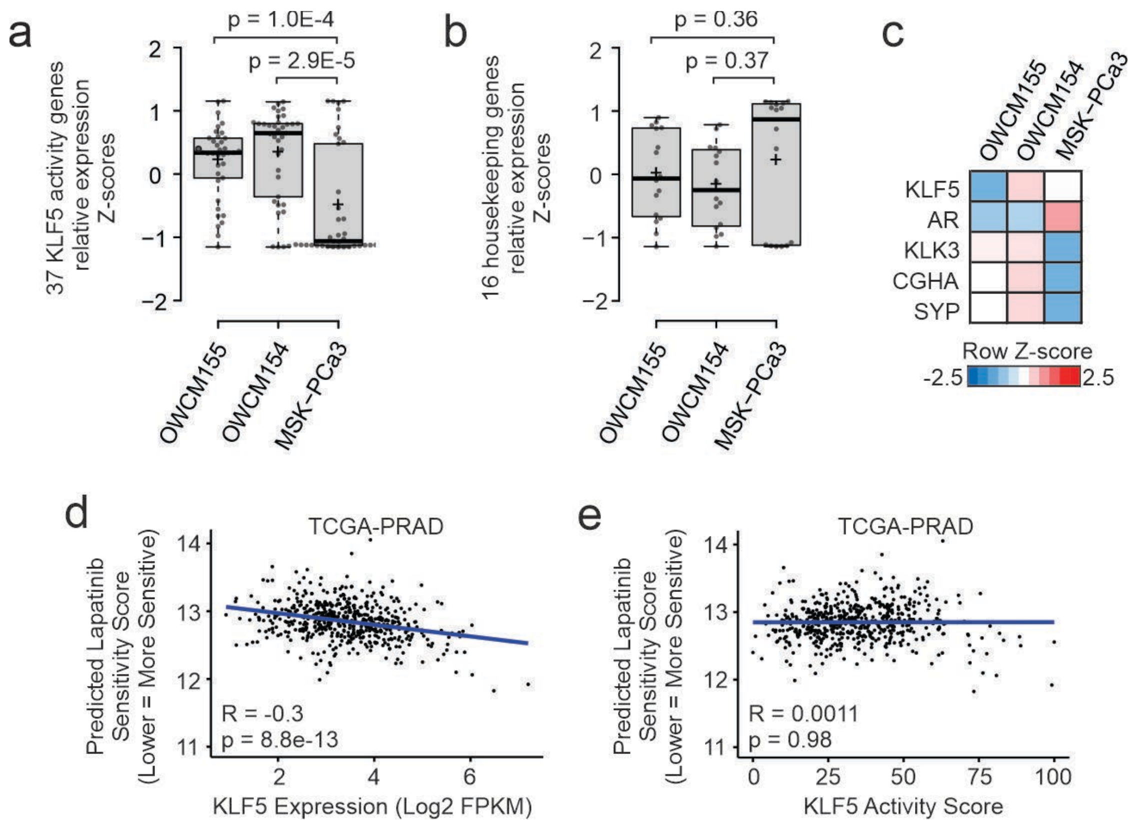

Supplementary Figure 18. Correlations of KLF5 Expression and Activity with Predicted Lapatinib Sensitivities. **a** Relative expression of 37 genes in the KLF5 activity score in NEPC patient-derived organoids (OWCM155 and OWCM154) and CRPC adenocarcinoma patient-derived organoids (MSK-PCa3). Bounds of boxes are lower/upper quartiles with median as a bold horizontal line and mean as a "+" sign, whiskers show range from minima to maxima; dots are the Z-score for each of the 37 genes in the KLF5 activity score. P-values are unadjusted from two-sided unpaired t-tests. **b** Relative expression of 16 housekeeping genes, with boxplots and t-tests as defined in a. **c** Z-scores of individual gene expression in indicated samples. **d, e** Ridge regression models trained on high-throughput cancer cell line drug screens were used to predict lapatinib sensitivity in prostate tumors from RNA-seq data generated by The Cancer Genome Atlas Prostate Adenocarcinoma Study (TCGA-PRAD). The predicted lapatinib sensitivity score was plotted vs. **a** KLF5 expression in Log2 FPKM (fragments per kilobase per million fragments mapped) or **b** KLF5 activity determined from summative z-scores of 37 KLF5 target genes. Pearson correlation coefficients (r) and 2-tailed p-values for r≠0 are shown.

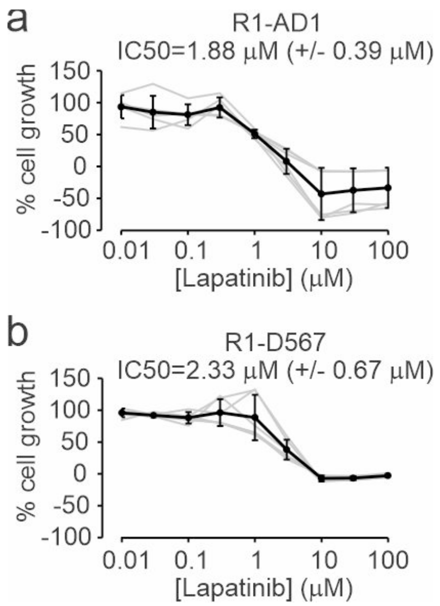

Supplementary Figure 19. Sensitivity of R1-AD1 and R1-D567 cells to lapatinib. a R1-AD1 and **b** R1-D567 cells were cultured in androgen-replete growth medium supplemented with indicated concentrations of lapatinib. Cells were subjected to 2-dimensional growth assays by fixing and crystal violet staining at start of treatment (day 0) and after 6 days treatment. Light gray lines represent growth data from individual biological replicate experiments (n=9) and dark lines represent the mean +/- 95% CI. Drug concentrations that resulted in 50% growth inhibition (IC50) were calculated from each biological replicate (n=9). The listed IC50 represents the mean +/- 95% CI.

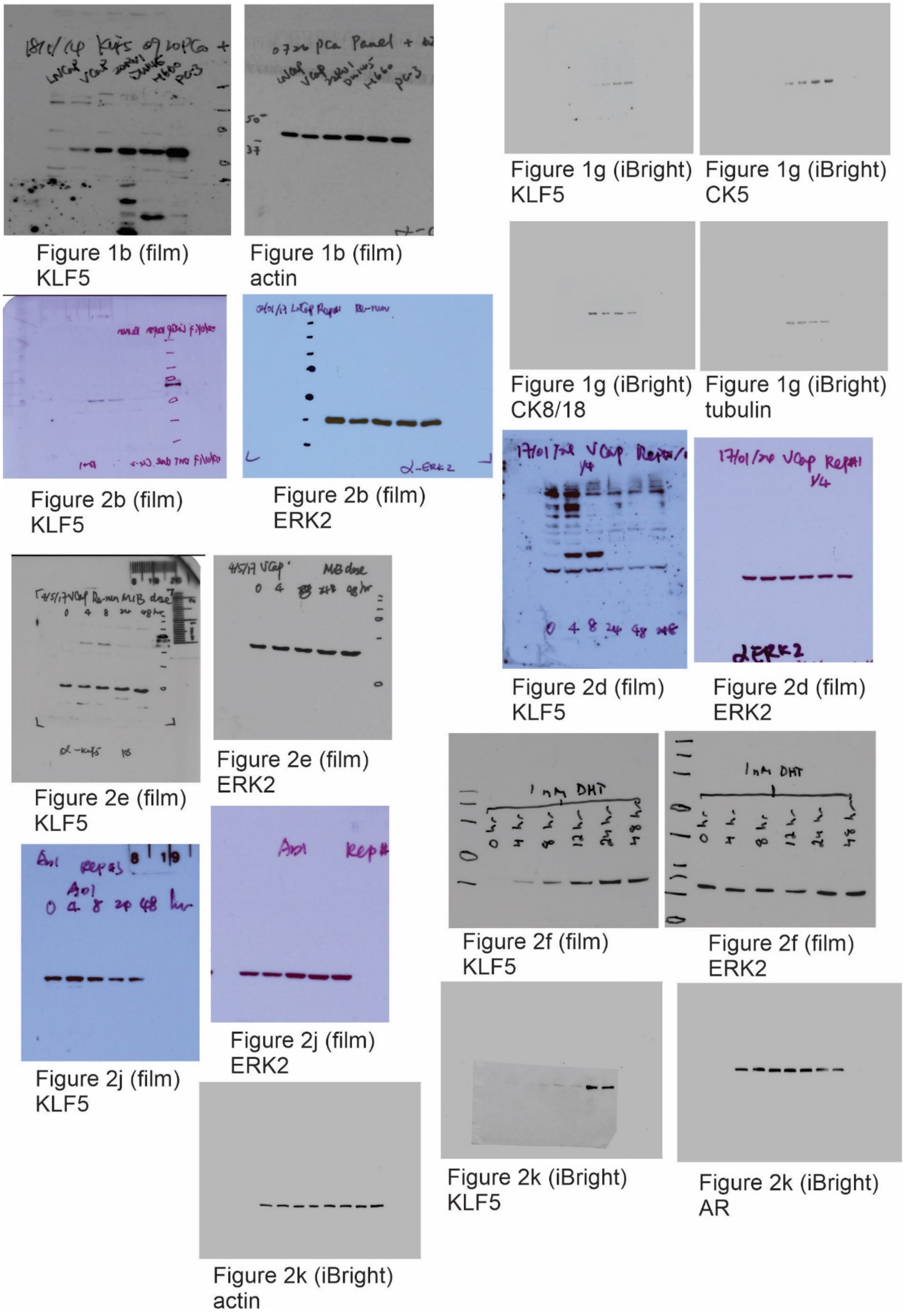

Supplementary Figure 20. Full western blot images for Figures 1&2

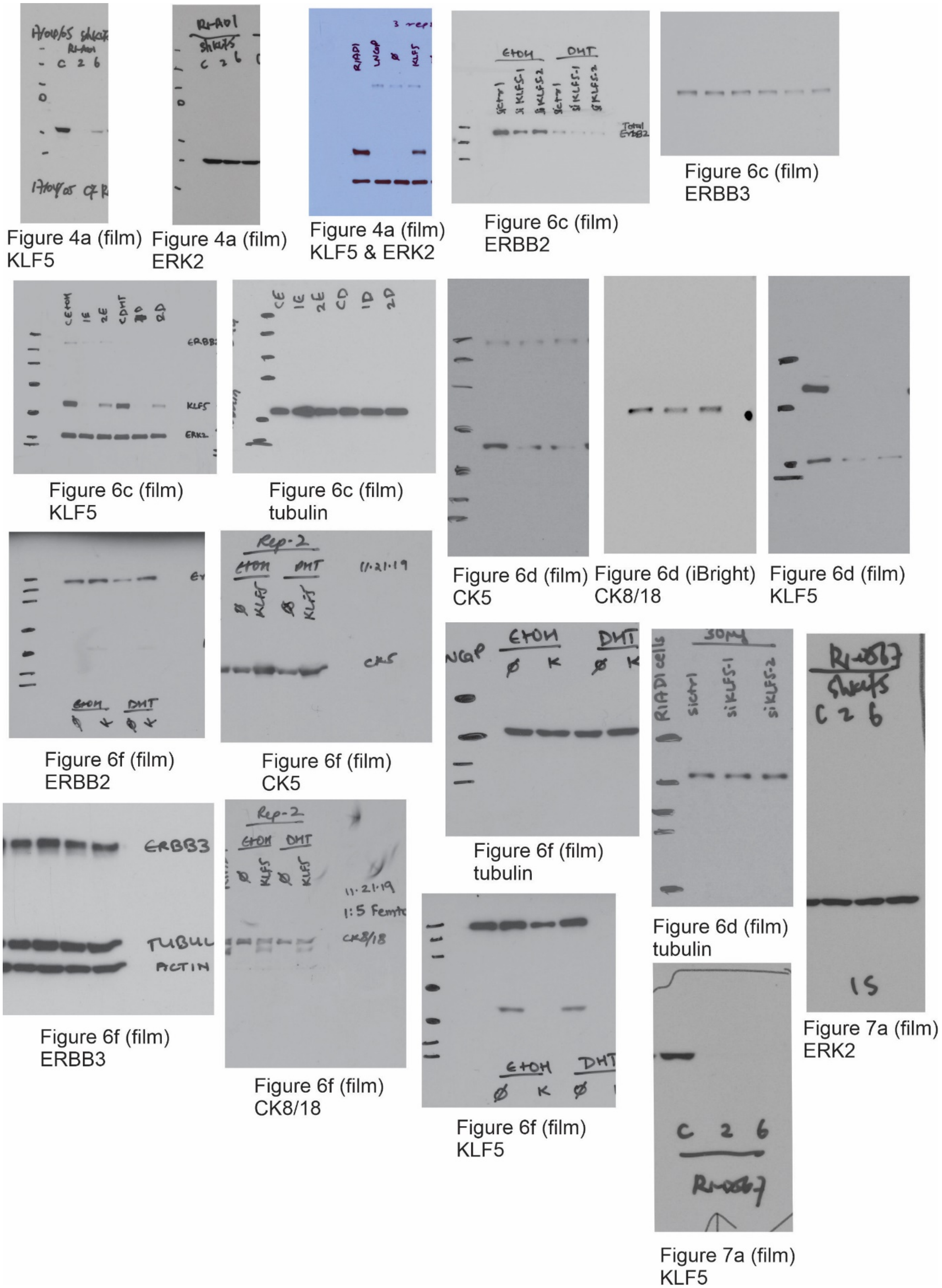

Supplementary Figure 21. Full western blot images for Figures 3-6

Supplementary References

1 Uphoff, C. C., Denkmann, S. A. & Drexler, H. G. Treatment of mycoplasma contamination in cell cultures with Plasmocin. *Journal of biomedicine & biotechnology* **2012**, 267678 (2012).

2 Chan, S. C., Li, Y. & Dehm, S. M. Androgen receptor splice variants activate AR target genes and support aberrant prostate cancer cell growth independent of the canonical AR nuclear localization signal. *J Biol Chem* **287**, 19736-19749 (2012).

3 Cerami, E. *et al.* The cBio cancer genomics portal: an open platform for exploring multidimensional cancer genomics data. *Cancer discovery* **2**, 401-404 (2012).

4 Gao, J. *et al.* Integrative analysis of complex cancer genomics and clinical profiles using the cBioPortal. *Sci Signal* **6**, pl1 (2013).

5 Robinson, D. *et al.* Integrative clinical genomics of advanced prostate cancer. *Cell* **161**, 1215-1228 (2015).

6 Kumar, A. *et al.* Substantial interindividual and limited intraindividual genomic diversity among tumors from men with metastatic prostate cancer. *Nat Med* **22**, 369-378 (2016).

7 Subramanian, A. *et al.* Gene set enrichment analysis: a knowledge-based approach for interpreting genome-wide expression profiles. *Proc Natl Acad Sci U S A* **102**, 15545-15550 (2005).

8 Abida, W. *et al.* Genomic correlates of clinical outcome in advanced prostate cancer. *Proc Natl Acad Sci U S A* **116**, 11428-11436 (2019).

9 Ashburner, M. *et al.* Gene ontology: tool for the unification of biology. The Gene Ontology Consortium. *Nat Genet* **25**, 25-29 (2000).

10 The Gene Ontology, C. The Gene Ontology Resource: 20 years and still GOing strong. *Nucleic Acids Res* **47**, D330-D338 (2019).

11 Wang, L. *et al.* A prospective genome-wide study of prostate cancer metastases reveals association of wnt pathway activation and increased cell cycle proliferation with primary resistance to abiraterone acetate-prednisone. *Annals of oncology : official journal of the European Society for Medical Oncology / ESMO* **29**, 352-360 (2018).

12 Network, C. G. A. R. The Molecular Taxonomy of Primary Prostate Cancer. *Cell* **163**, 1011-1025 (2015).

13 Goldman, M. *et al.* The UCSC Xena platform for public and private cancer genomics data visualization and interpretation. *bioRxiv*, 326470 (2019).

14 Basu, A. *et al.* An interactive resource to identify cancer genetic and lineage dependencies targeted by small molecules. *Cell* **154**, 1151-1161 (2013).

15 Rees, M. G. *et al.* Correlating chemical sensitivity and basal gene expression reveals mechanism of action. *Nature chemical biology* **12**, 109-116 (2016).

16 Seashore-Ludlow, B. *et al.* Harnessing Connectivity in a Large-Scale Small-Molecule Sensitivity Dataset. *Cancer discovery* **5**, 1210-1223 (2015).

17 Barretina, J. *et al.* The Cancer Cell Line Encyclopedia enables predictive modelling of anticancer drug sensitivity. *Nature* **483**, 603-607 (2012).

18 Geeleher, P., Cox, N. J. & Huang, R. S. Clinical drug response can be predicted using baseline gene expression levels and in vitro drug sensitivity in cell lines. *Genome Biol* **15**, R47 (2014).

19 Friedman, J., Hastie, T. & Tibshirani, R. Regularization Paths for Generalized Linear Models via Coordinate Descent. *J Stat Softw* **33**, 1-22 (2010).

20 Durinck, S. *et al.* BioMart and Bioconductor: a powerful link between biological databases and microarray data analysis. *Bioinformatics* **21**, 3439-3440 (2005).

21 Durinck, S., Spellman, P. T., Birney, E. & Huber, W. Mapping identifiers for the integration of genomic datasets with the R/Bioconductor package biomaRt. *Nature protocols* **4**, 1184-1191 (2009).
