# Supplementary material for: Opposing transcriptional programs of KLF5 and AR emerge during therapy for advanced prostate cancer
Source: Nat Commun. 2021 Nov 4;12:6377. doi: 10.1038/s41467-021-26612-1 (PMC8568894; doi:10.1038/s41467-021-26612-1)
Supplement: Supplementary file 2 — Description of Additional Supplementary Files [file 41467_2021_26612_MOESM2_ESM.pdf]

Description of Additional Supplementary Files

File Name: Supplementary Data 1
Description: Gene signatures

File Name: Supplementary Data 2
Description: Genes differentially expressed between shCON vs. shKLF5 conditions in DHT-treated R1-AD1 cells.

File Name: Supplementary Data 3
Description: Genes differentially expressed between shCON vs. shKLF5 conditions in ETH-treated R1-AD1 cells.

File Name: Supplementary Data 4
Description: Genes differentially expressed between DHT vs. ETH conditions in R1-AD1 cells infected with shCON lentivirus.

File Name: Supplementary Data 5
Description: Genes differentially expressed between DHT vs. ETH conditions in R1-AD1 cells infected with shKLF5 lentivirus

File Name: Supplementary Data 6
Description: Genes differentially expressed between shCON vs. shKLF5 conditions in R1-D567 cells.
