# Supplementary material for: Opposing transcriptional programs of KLF5 and AR emerge during therapy for advanced prostate cancer
Source: Nat Commun. 2021 Nov 4;12:6377. doi: 10.1038/s41467-021-26612-1 (PMC8568894; doi:10.1038/s41467-021-26612-1)
Supplement: Supplementary file 9 — Reporting Summary [file 41467_2021_26612_MOESM9_ESM.pdf]

nature portfolio

Corresponding author(s): Scott Dehm

Last updated by author(s): Sep 16, 2021

Reporting Summary

Nature Portfolio wishes to improve the reproducibility of the work that we publish. This form provides structure for consistency and transparency in reporting. For further information on Nature Portfolio policies, see our Editorial Policies and the Editorial Policy Checklist.

Statistics

For all statistical analyses, confirm that the following items are present in the figure legend, table legend, main text, or Methods section.

n/a	Confirmed	
☐	☒	The exact sample size (n) for each experimental group/condition, given as a discrete number and unit of measurement
☐	☒	A statement on whether measurements were taken from distinct samples or whether the same sample was measured repeatedly
☐	☒	The statistical test(s) used AND whether they are one- or two-sided<br>*Only common tests should be described solely by name; describe more complex techniques in the Methods section.*
☐	☒	A description of all covariates tested
☐	☒	A description of any assumptions or corrections, such as tests of normality and adjustment for multiple comparisons
☐	☒	A full description of the statistical parameters including central tendency (e.g. means) or other basic estimates (e.g. regression coefficient) AND variation (e.g. standard deviation) or associated estimates of uncertainty (e.g. confidence intervals)
☐	☒	For null hypothesis testing, the test statistic (e.g. F, t, r) with confidence intervals, effect sizes, degrees of freedom and P value noted<br>*Give P values as exact values whenever suitable.*
☒	☐	For Bayesian analysis, information on the choice of priors and Markov chain Monte Carlo settings
☒	☐	For hierarchical and complex designs, identification of the appropriate level for tests and full reporting of outcomes
☐	☒	Estimates of effect sizes (e.g. Cohen's d, Pearson's r), indicating how they were calculated

Our web collection on statistics for biologists contains articles on many of the points above.

Software and code

Policy information about availability of computer code

Data collection	Bio-Rad CFX manager software, Nikon NIS Elements D 4.00.12 software
Data analysis	ImageJ, GraphPad Prism 6, bwa (v. 0.7.17 for cell line data, version 0.5.10 for clinical data), IGV (version 2.3.93), bedtools (v. 2.27.1), Macs2 (v. 2.1.1), HiSat2 (v. 2.1.0), Subread (v. 1.4.6), edgeR (v. 3.20.9) , GSEA java program (v. 3.0), FASTQC (v0.11.8), Trim Galore (v0.6.0), TopHat (v2.0.6), featureCounts 2 package (v1.5.2), R packages: readxl version 1.3.1, progress version 1.2.2, glmnet version 3.0.1, sva version 3.34.0, doParallel version 1.0.15, biomaRt version 2.32.1

For manuscripts utilizing custom algorithms or software that are central to the research but not yet described in published literature, software must be made available to editors and reviewers. We strongly encourage code deposition in a community repository (e.g. GitHub). See the Nature Portfolio guidelines for submitting code & software for further information.

Data

Policy information about availability of data

All manuscripts must include a data availability statement. This statement should provide the following information, where applicable:

- Accession codes, unique identifiers, or web links for publicly available datasets
- A description of any restrictions on data availability
- For clinical datasets or third party data, please ensure that the statement adheres to our policy

ChIP-seq and RNA-seq data generated for this study are available at NCBI GEO accession GSE148808 as described in the Methods section.

Field-specific reporting

Please select the one below that is the best fit for your research. If you are not sure, read the appropriate sections before making your selection.

☒ Life sciences ☐ Behavioural & social sciences ☐ Ecological, evolutionary & environmental sciences

For a reference copy of the document with all sections, see nature.com/documents/nr-reporting-summary-flat.pdf

Life sciences study design

All studies must disclose on these points even when the disclosure is negative.

Sample size	Individual data points from replicate experiments are shown in all figures. All figure legends describe the sample size for individual graphs. For RNA-seq we had n=3 biological replicates which is standard for RNA-seq analysis of cell lines. For ChIP-seq we had two replicates for each transcription factor as per ENCODE guidelines.
Data exclusions	No data were excluded from the study.
Replication	As mentioned with all relevant figures, sufficient technical and biological replicates of the experiments were performed and the data is reproducible using the methods and models described by the authors.
Randomization	N/A
Blinding	N/A

Reporting for specific materials, systems and methods

We require information from authors about some types of materials, experimental systems and methods used in many studies. Here, indicate whether each material, system or method listed is relevant to your study. If you are not sure if a list item applies to your research, read the appropriate section before selecting a response.

Materials & experimental systems

n/a	Involved in the study	
☐	☒	Antibodies
☐	☒	Eukaryotic cell lines
☒	☐	Palaeontology and archaeology
☒	☐	Animals and other organisms
☒	☐	Human research participants
☒	☐	Clinical data
☒	☐	Dual use research of concern

Methods

n/a	Involved in the study	
☐	☒	ChIP-seq
☒	☐	Flow cytometry
☒	☐	MRI-based neuroimaging

Antibodies

Antibodies used	AR (Santa Cruz, N-20, sc-816, 1:1000),<br>AR (Cell Marque, SP107, 200R-16, 1:4000)<br>b-Actin (Santa Cruz, C4, sc-47778, 1:3000)<br>Tubulin (Santa Cruz, B-5-1-2, sc-23948, 1:3000)<br>ERK2 (Santa Cruz, D-2, sc-1647, 1:4000)<br>KLF5 (Santa Cruz, G-7, sc-398470, 1:2000)<br>ERK-2 (Santa Cruz, D-2, sc-1647)<br>ERBB2 (Cell Signaling, 29D8, #2165, 1:1000)<br>ERBB3 (Cell signaling, D22C5, #12708, 1:1000)<br>CK5 (Sigma, SAB4501651, 1:1000)<br>CK8/18 (Leica Biosystems, NCL-L-5D3, 1:3000)
Validation	Most of the antibodies have been pre-validated by the manufacturer and previously reported in the literature. All antibodies were validated in the lab by band specificity and product size in western blot experiments. Antibodies for AR and KLF5 were further validated for quantitative detection of decreases in protein expression following knock-down with gene-specific siRNAs and/or shRNAs.

Eukaryotic cell lines

Policy information about cell lines

Cell line source(s)	Cell lines purchased from American Type Culture Collection (ATCC): LNCaP, VCaP, 22RV1, DU145, PC3 and NCI-H660 cells.<br>Cell line LNCaP95 were a gift from Jun Luo (Johns Hopkins University).<br>LNCaP sublines (V16D, 49F, 42D) are derived from xenografts in castrated or castrated/enzalutamide-treated mice as have been described in Reference 19 of this manuscript.<br>Cell Lines R1AD1 and R1D567 have also been previously described in Reference 45 of this manuscript.
Authentication	As described in our Supplemental materials and methods: "ATCC ensures authenticity of LNCaP, VCaP, 22RV1, DU145, PC3 and NCI-H660 cell lines using short tandem repeat analysis. Authentication of the R1-AD1 cell line was performed by sequence-based validation of an AR H874Y point mutation and authentication of the R1-D567 cell line was performed by PCR-based validation of a targeted genomic deletion of AR exons 5-7. Authentication of LNCaP-derived sub-lines was performed by sequence-based validation of an AR T878A point mutation."
Mycoplasma contamination	As mentioned in our Supplemental materials and methods: "Aliquots of cell culture supernatants from cells in active culture were evaluated regularly for mycoplasma contamination using a PCR-based method as described (Uphoff CC, Denkmann SA, Drexler HG. Treatment of mycoplasma contamination in cell cultures with Plasmocin. Journal of Biomedicine & Biotechnology 2012;2012:267678. doi:10.1155/2012/267678). All cell line experiments were performed within 2-3 months of resuscitation of frozen cell stocks prepared within 3 passages of receipt."
Commonly misidentified lines<br>(See ICLAC register)	N/A

ChIP-seq

Data deposition

☒ Confirm that both raw and final processed data have been deposited in a public database such as GEO.

☐ Confirm that you have deposited or provided access to graph files (e.g. BED files) for the called peaks.

Data access links<br>*May remain private before publication.*	ChIP-seq and RNA-seq data generated for this study are available at NCBI GEO accession GSE148808. To access go to the link: https://www.ncbi.nlm.nih.gov/geo/query/acc.cgi?acc=GSE148808. Enter token gzadusakhtgtdkd into the box.
Files in database submission	The files in GEO accession GSE148808 are:<br>GSM4481511 input DNA_R1-AD1 rep 1<br>GSM4481512 input DNA_R1-AD1 rep 2<br>GSM4481513 KLF5_R1-AD1_DHT_ChIP-seq rep1<br>GSM4481514 KLF5_R1-AD1_DHT_ChIP-seq rep2<br>GSM4481515 KLF5_R1-AD1_ETH_ChIP-seq rep1<br>GSM4481516 KLF5_R1-AD1_ETH_ChIP-seq rep2<br>GSM4481517 input DNA_R1-D567 rep 1<br>GSM4481518 input DNA_R1-D567 rep 2<br>GSM4481519 KLF5_R1-D567_ChIP-seq rep1<br>GSM4481520 KLF5_R1-D567_ChIP-seq rep2<br>GSE148807_AD1KLF5DHTmerged.narrowPeak.gz 532.6 Kb (http) NARROWPEAK<br>GSE148807_AD1KLF5ETHmerged.narrowPeak.gz 398.3 Kb (http) NARROWPEAK<br>GSE148807_AD1_DHT_KLF5.bigwig 299.2 Mb (http) BIGWIG<br>GSE148807_AD1_ETH_KLF5.bigwig 275.3 Mb (http) BIGWIG<br>GSE148807_D567KLF5merged.narrowPeak.gz 128.4 Kb (http) NARROWPEAK<br>GSE148807_D567_ETH_KLF5.bigwig 318.8 Mb (http) BIGWIG
Genome browser session<br>(e.g. UCSC)	no longer applicable

Methodology

Replicates	2 biological replicates for each condition
Sequencing depth	As described below, the range of read depths that we achieved were 20,504,078-24,518,952 reads per sample:<br>Sample : PF Clusters<br>AD1INPUT-1 : 20,504,078<br>AD1INPUT2 : 23,818,129<br>AD1KLF5DHT-1 : 21,594,692<br>AD1KLF5DHT-2 : 23,515,452<br>AD1KLF5ETH-1 : 21,102,803<br>AD1KLF5ETH-2 : 21,528,443<br>D567INPUT-1 : 24,518,952<br>D567INPUT-2 : 21,359,083

	D567KLF5-1 : 24,445,340<br>D567KLF5-2 : 22,614,242
Antibodies	AR antibody (Santa Cruz, N-20, sc-816, 1:50)<br>BTEB-2 or KLF5 antibody (Santa Cruz, H-300, sc-22797, 1:50)
Peak calling parameters	KLF5 and AR ChIP-seq data sets were mapped using bwa (v. 0.7.17) against the human (hg19) reference genome. Mapped data were filtered to keep only reads with a mapq = 1 using samtools (v.1.7). Bam files were filtered using bedtools (v. 2.27.1) to remove any ENCODE blacklisted regions (ENCFF001TDO.bed). Macs2 (v. 2.1.1) was used for peak calling using paired treatment and control bam files. Data from treatment replicates were merged to create bam files and bigwigs for visualization. We used the bedtools 'intersect' and 'subtract' functions to obtain final peak sets. For the peak sets derived from KLF5 ChIP-Seq, we reduced the number of peaks by finding the intersect across all replicates. For the peak sets derived from AR ChIP-seq, we used the AR ChIP-seq replicate that yielded the most peaks. H3K27ac ChIP-seq data were analyzed separately and are from a different study, these data were mapped with bwa and visualizations were created using IGV.
Data quality	All peaks that were called with Macs2 in this study (for AR and KLF5) were below the FDR of 0.05% and were called using the sample and Input control files together with Macs2 callpeak. We did not change the default settings to include a fold-change cutoff.
Software	bwa (v. 0.7.17), samtools (v.1.7), bedtools (v. 2.27.1), Macs2 (v. 2.1.1), IGV (v. 2.8.2)
